# Function Analysis of P450 and GST Genes to Imidacloprid in *Aphis craccivora* (Koch)

**DOI:** 10.3389/fphys.2020.624287

**Published:** 2021-01-20

**Authors:** Yuan-Xue Yang, Rong-Hua Lin, Zhuo Li, Ai-Yu Wang, Chao Xue, Ai-Ling Duan, Ming Zhao, Jian-Hua Zhang

**Affiliations:** ^1^Cotton Research Center, Shandong Academy of Agricultural Sciences, Jinan, China; ^2^Institute for the Control of Agrochemicals, Ministry of Agriculture and Rural Affairs, Beijing, China; ^3^Institute of Plant Protection, Jiangsu Academy of Agricultural Sciences, Nanjing, China

**Keywords:** Aphis craccivora, P450 genes, GST genes, imidacloprid, expression induction, RNA interference

## Abstract

*Aphis craccivora* (Koch) is an economically important pest that affects legumes in worldwide. Chemical control is still the primary efficient method for *A. craccivora* management. However, the mechanism underlying insecticide resistance in *A. craccivora* has not been elucidated. A previous study observed that piperonyl butoxide (PBO) and diethyl maleate (DEM) significantly synergized imidacloprid in *A. craccivora* field populations, indicating that cytochrome P450 (P450) and glutathione S-transferase (GST) genes may play pivotal roles in imidacloprid resistance. In this study, 38 P450 genes and 10 GST genes were identified in *A. craccivora* through transcriptomic analysis. The expression levels of these P450 and GST genes were measured in susceptible (SUS) strains of *A. craccivora* under imidacloprid treatment with LC_15_, LC_50_, and LC_85_ doses. The expression levels of *CYP18A1*, *CYP6CY21*, *CYP6DA1*, *CYP6DA2*, *CYP4CJ1*, *CYP4CJ2*, and *CYP380C6* were up-regulated in the three treatments. Most of these genes belong to CYP3 and CYP4 Clans. In addition, the expression levels of all P450 and GST genes in *A. craccivora* were also measured in the Juye (JY) and Linqing (LQ) field populations. The expression levels of *CYP6DA2*, *CYP4CJ1*, and *CYP380C6* were up-regulated in the SUS strain after imidacloprid treatment at three doses, and these genes were overexpressed in the JY population. Furthermore, the sensitivity of *A. craccivora* to imidacloprid was significantly increased after knockdown of *CYP380C6* and *CYP6DA2* through RNA interference. These results may help to elucidate the mechanisms underlying of imidacloprid resistance in *A. craccivora*.

## Introduction

The *Aphis craccivora* (Koch), is one of the most important worldwide, affecting multiple legumes, such as pea, cowpea, and peanut. *A. craccivora* causes serious yield losses in legumes by sucking the leaf, bud, and flower sap. In addition, *A. craccivora* can transmit two major plant viruses, bean leaf roll virus and faba bean necrotic yellows virus, which seriously affects the yield and quality of legumes (Li et al., 2013a). Insecticides, including pyrethroids, organophosphates, and neonicotinoids, are currently the primary management tool or controlling *A. craccivora* (Zhang et al., 2015; Fouad et al., 2016). However, due to the extensive and recurrent use of these insecticides, field populations of *A. craccivora* have developed insecticide resistance (Li et al., 2013a; Abd-Ella, 2014).

The molecular mechanism underlying insecticide resistance primarily includes the reduction of target insensitivity and the enhancement of metabolic detoxification. Metabolic resistance to insecticides primarily due to the overexpression of three enzymes, including cytochrome P450 monooxygenases (P450s), glutathione S-transferases (GSTs), and carboxylesterase (CarEs; Li et al., 2007). P450s are a multi-gene superfamily which can participate in the synthesis and metabolism of endogenous compounds, and able to metabolize many types of xenobiotics, such as insecticides and plant toxins (Scott and Wen, 2001; Feyereisen, 2005, 2011). The P450 genes are primarily divided into four Clans: CYP2, CYP3, CYP4, and Mitochondrial (Mito) Clan (Feyereisen, 1999). P450s are involved in imidacloprid resistance in several pests; these genes include *CYP6AY1*, *CYP6ER1*, *CYP6CW1*, and *CYP4CE1* in *Nilaparvata lugens*, *CYP6CM1* in *Bemisia tabaci*, *CYP9M10* in *Culex quinquefasciatus*, and *CYP6D1* in *Musca domestica* (Liu and Scott, 1998; Karunker et al., 2008; Bass et al., 2011; Nauen et al., 2013; Zimmer et al., 2014). Also, GSTs, which are involved in the detoxification of exogenous toxins and intracellular transport, are important conjugation enzymes (De and Hemingway, 2010). The GST genes can be divided into seven classes, including Delta, Epsilon, Omega, Sigma, Theta, Zeta, and Microsomal (Enayati et al., 2005). In *N. lugens*, GSTs could be utilized as antioxidant defense agents to confer resistance to pyrethroid (Vontas et al., 2001). The Delta and Epsilon classes are insect-specific, playing important roles in xenobiotic detoxification (Che-Mendoza et al., 2009). The expression of *AgGSTe2*, which can metabolize DDT, was more than 5.0-fold in the DDT-resistant strain of *Anopheles gambiae* compared with the susceptible (SUS) strain (Ranson et al., 2000). Finally, CarEs play important roles in the degradation of insecticides *via* hydrolysis and catalysis (Li et al., 2007). In *Aphis gossypii*, the increased expression of CarEs was related to resistance to omethoate (Gong et al., 2014).

Imidacloprid is a representative neonicotinoid insecticide and has been extensively utilized in the management of piercing-sucking pests, such as aphids, planthoppers, leafhoppers, and thrips (Choi et al., 2001; Nauen and Denholm, 2010; Wang et al., 2018). However, different levels of imidacloprid resistance have been developed in different piercing-sucking pests due to the wide application of this insecticide (Karunker et al., 2008; Wen et al., 2009; Andrei et al., 2010; Cui et al., 2016). The populations of *A. gossypii*, such as Langfang (Hebei Province, China), Akesu (Xinjiang Province, China), Dezhou (Shandong Province, China), and Kuitun (Xinjiang Province, China), exhibited moderate levels of resistance to imidacloprid (Cui et al., 2016). Six populations of *Myzus persicae* showed high-levels of resistance to imidacloprid in South China (Zhang and Zhou, 2014). In previous studies, the constitutive overexpression of detoxification genes has been demonstrated to be related to insecticide resistance (Li et al., 2007). In addition, the induction of detoxification gene expression by insecticides is a rapid and effective method to identify key genes related to metabolic resistance, especially in the early stages of resistance development (Terriere, 1984; Zhang et al., 2016a).

In previous studies, we found that the resistance ratio of imidacloprid in Juye (JY, Shandong Province, China) and Linqing (LQ, Shandong Province, China) populations was 8.32- and 3.68-fold, exhibiting low resistance and sensitivity levels, respectively (Yang et al., 2020). Furthermore, piperonyl butoxide (PBO) and diethyl maleate (DEM) significantly synergized with imidacloprid in the field populations of *A. craccivora*. These results indicated that the P450s and GSTs may play important roles in imidacloprid tolerance in field populations of *A. craccivora*. In this study, the P450 and GST genes were identified *via* transcriptome analysis, and the changes in the expression levels of these genes were detected in the *A. craccivora* SUS strain under imidacloprid treatment. The expression levels of the P450 and GST genes were also detected in the field populations of *A. craccivora*. Furthermore, the functions of the up-regulated genes in imidacloprid treatment were verified through RNA interference (RNAi). These results may establish a foundation for further research exploring the mechanism underlying imidacloprid resistance in *A. craccivora*.

## Materials and Methods

### Insects

The susceptible (SUS) strain of *A. craccivora* was obtained from the China Southern Pesticide Creation Center (Shanghai, China) and reared in the laboratory without any contact with insecticides. The SUS strain of *A. craccivora* was collected from Shanghai (China) in 1980. The field populations of *A. craccivora* were collected from Linqing (LQ, Shandong Province, China, 115.702478E, 36.81416N) and Juye (JY, Shandong Province, China, 116.146121E, 35.225549N) in 2019. Insects were reared on *Vicia faba* plants at 25 ± 1°C with 70–80% humidity and a 16 h light: 8 h dark photoperiod.

### Insecticides and Chemicals

Imidacloprid technical (96.7%) was provided by Shandong Lukang Biological Pesticide CO., Ltd. Dimethl sulfoxide (DMSO) and Triton X-100 were purchased from Sigma-Aldrich (St. Louis, MO, USA).

### Expression Induction

For expression induction, brachypterous *A. craccivora* adults were treated with imidacloprid at doses of LC_15_ (0.02 mg/L), LC_50_ (0.36 mg/L), and LC_85_ (4.2 mg/L). The experiment was performed according to the leaf-dipping method (Fouad et al., 2016). Imidacloprid was dissolved in DMSO and subsequently diluted in distilled water containing 0.05% Triton X-100 to generate a given concentration. *Vicia faba* seeding with brachypterous *A. craccivora* adults were immersed in imidacloprid for 5 s. Also, seedlings with *A. craccivora* brachypterous adult dipped in distilled water containing 0.05% Triton X-100 and 1% DMSO as a control. Filter paper was used to absorb excess liquid and transfer the *Vicia faba* seeding to the feeding box. All insects were reared at 25 ± 1°C, with 70–80% humidity and 16 h light: 8 h dark photoperiod. An aphid was considered dead if it was incapable of coordinated forward movement. Survival insects were collected at 48 h after imidacloprid application. The experiment included three replicates, and every replicate contained 30 adults. The insects were frozen in liquid nitrogen and stored at −80°C.

### RNA Isolation, Purification, and Quantification

Total RNA was extracted using TRIzol reagent (Life Technologies, USA) following the manufacturer’s instructions. Spectrophotometry (NanoDrop 2000, Thermo Scientific) and agarose gel electrophoresis were employed to detect the quantity and quality of the RNAs, respectively. The RNA integrity was assessed by Agilent 2100 with RIN number >7.0.

### cDNA Library Construction

Poly (A) RNA was purified from total RNA (10 μg) using poly T oligo attached magnetic beads using two rounds of purification. Then the poly (A) RNA was fragmented into small pieces using divalent cations under high temperature. Then the cleaved RNA fragments were reverse transcribed to create the cDNA, which were next used to synthesize U labeled second stranded DNAs with *Escherichia coli* DNA polymerase I, RNase H and dUTP. An A base was then added to the blunt ends of each strand, preparing them for ligation to the indexed adapters. Each adapter contains a T base overhang for ligating the adapter to the A tailed fragmented DNA. Single or dual index adapters were ligated to the fragments, and size selection was performed with AMPureXP beads. After the heat labile UDG enzyme treatment of the U labeled second stranded DNAs, the ligated products were amplified with PCR by the following conditions: initial denaturation at 95°C for 3 min; 8 cycles of denaturation at 98°C for 15 s, annealing at 60°C for 15 s, and extension at 72°C for 30 s; and then final extension at 72°C for 5 min. The average insert size for the final cDNA library was 300 bp (±50 bp). At last, we performed the 150 bp paired end sequencing on an Illumina Hiseq 6000 (LC Bio, China) following the vendor’s recommended protocol.

### Bioinformatic Analysis

Cutadapt and perl scripts in house were used to remove the reads that contained adaptor contamination, low quality bases and undetermined bases (Martin, 2011). The sequence quality was verified using FastQC.^1^ Trinity 2.4.0 was performed to *de novo* assembly of the transcripts (Grabherr et al., 2011). The longest transcript in the cluster was chosen as the representative gene sequence. All assembled transcripts were aligned against the non-redundant (Nr) protein database,^2^ Gene Ontology (GO),^3^ SwissProt,^4^ Kyoto Encyclopedia of Genes and Genomes (KEGG),^5^ and eggnog databases using DIAMOND with a threshold value of *E* < 10^−5^ (Buchfink et al., 2015).^6^

### Identification of P450 and GST Genes and Phylogenetic Analysis

The sequences of P450 and GST genes were searched in the transcriptome of *A. craccivora* according to the annotation information. After removing the short (<300-bp) and repeated sequences, the remaining sequences were aligned in the NCBI database by BLASTx (value of *E* < 10^−5^).^7^ MEGA 5.05 was employed to construct the phylogenetic trees using the neighbor joining method based on the amino acid sequences in *A. craccivora* and other insects (Tamura et al., 2011). Information on the other insects in the phylogenetic trees was listed in Supplementary Table S1. The P450 genes were named by the P450 nomenclature committee (David R. Nelson, Department of Molecular Sciences, University of Tennessee, Memphis, TN, USA). The GST genes were named by phylogenetic analysis and the BLASTx results. The GenBank accession numbers of these P450 and GST genes were deposited in NCBI and the detailed information is presented in Supplementary Table S2.

### Sequence Verification

According to the sequence information obtained from the transcriptome, the specific primer for each gene was designed using Primer5 (Supplementary Table S2). Total RNA was extracted using TRIzol reagent (Life Technologies, USA) following the manufacturer’s instructions. cDNA was synthesized following the manufacturer’s instructions for HiScript III RT SuperMix for qPCR (Vazyme Biotech Co., Ltd.). 2 × Taq Plus Master Mix II (Vazyme Biotech Co., Ltd.) was used for PCR. The amplified products were validated by gel electrophoresis and sequenced at Sangon Biotech (Shanghai, Co., Ltd.).

### Detection of P450 and GST Genes by Quantitative Real-Time PCR

RT-qPCR was performed to detect the expression of P450 and GST genes at the mRNA level. A SYBR PrimeScript™ RT-PCR Kit (Takara, Japan) and IQ™ 5 multicolor real-time PCR detection system (BIO-RAD, USA) were utilized. Each experiment included three independent biological replications, and every biological replication included three technical replications. Every biological replication contained 15 insects. The relative expression of each gene was calculated by the 2^−*Δ*ΔCt^ method. *RPS8* (Ribosomal protein S8, GenBank No.: GAJW01000269) and *RPL14* (Ribosomal protein L14, GenBank No.: GAJW01000046) were used as the internal reference genes (Yang et al., 2015). The Geometric mean of two reference genes was considered as a normalizer (Gharbi et al., 2015). The specific primer of each gene for RT-qPCR were listed in Supplementary Table S2.

### RNA Interference

Double-stranded RNA (dsRNA) was synthesized using a T7 high yield transcription kit (Invitrogen, USA) according to the manufacturer’s instructions. The primers for dsRNA synthesis were listed in Supplementary Table S2. The rearing device used for this study were developed based on the methods of Mittler with some modifications (Mittler and Dadd, 1964). The dsRNA of target gene was added to the artificial diet (0.5 M sterile sucrose solution) at a concentration of 150 ng/μl. The artificial diet containing dsRNA-EGFP was employed as the control. The aphids were transferred onto the artificial diet for rearing. The experiments included three biological replications, and each replication included 30 aphids. After 48 h, the efficiency of the dsRNA knockdown of the three P450s expression was analyzed through RT-qPCR. To assess the sensitivity of the *A. craccivora* to imidacloprid, the brachypterous adults were treated with imidacloprid (LC_50_ dose) after knockdown of P450s in 48 h. The mortality of *A. craccivora* was checked after 48 h.

### Statistical Analysis

SPSS 20.0 (IBM Corporation, USA) was utilized to perform the statistical analysis. One-way analysis of variance (ANOVA) with the least significant difference (LSD) test was employed in comparing the gene expression levels. The value of *p* < 0.05 and value of *p* < 0.01 were regarded as significant and very significant differences, respectively.

## Results

Identification of P450 and GST Genes in *A. craccivora* Through Transcriptome

Approximately 54,778,308 raw reads were generated from Illumina sequencing of the *A. craccivora* cDNA library (Supplementary Table S3). After removing the low-quality reads, 52,576,182 valid reads were obtained. The clean reads were finally assembled into 39,048 transcripts with a mean length of 689 bp and an N50 length of 1852 bp (Supplementary Table S3). These unigenes were annotated against the NR, COG, KEGG and Swiss-Prot databases with value of *E* < 10^−5^. The highest match percentage was to *A. gossypii* (55.58%), followed by *Rhopalosiphum maidis* (11.98%), *Melanaphis sacchari* (5.1%), *Acyrthosiphon pisum* (4.34%), and *M. persicae* (3.06%; Supplementary Figure S1). To further elucidate the functions of these unigenes, GO assignments were utilized to classify unigenes into different functional groups according to GO category. Based on sequence homology, 10,383 unigenes were annotated and classified into one or more functional groups corresponding to the three biological processes. Ultimately, 14,860 annotation hits were aligned to biological process, 9,317 to cellular components, and 18,014 to molecular functions (Supplementary Figure S2). The transcriptome data has been deposited in the NCBI database (GEO accession: GSE161346).

P450 and GST sequences were identified by a BLAST search against the transcriptomic database of *A. craccivora*. The complete coding region was confirmed by ORF finder and protein BLAST results (Supplementary Table S4). These identified genes were classified into different subfamilies *via* alignment between *A. craccivora* and other insect species.

**Figure 1:**
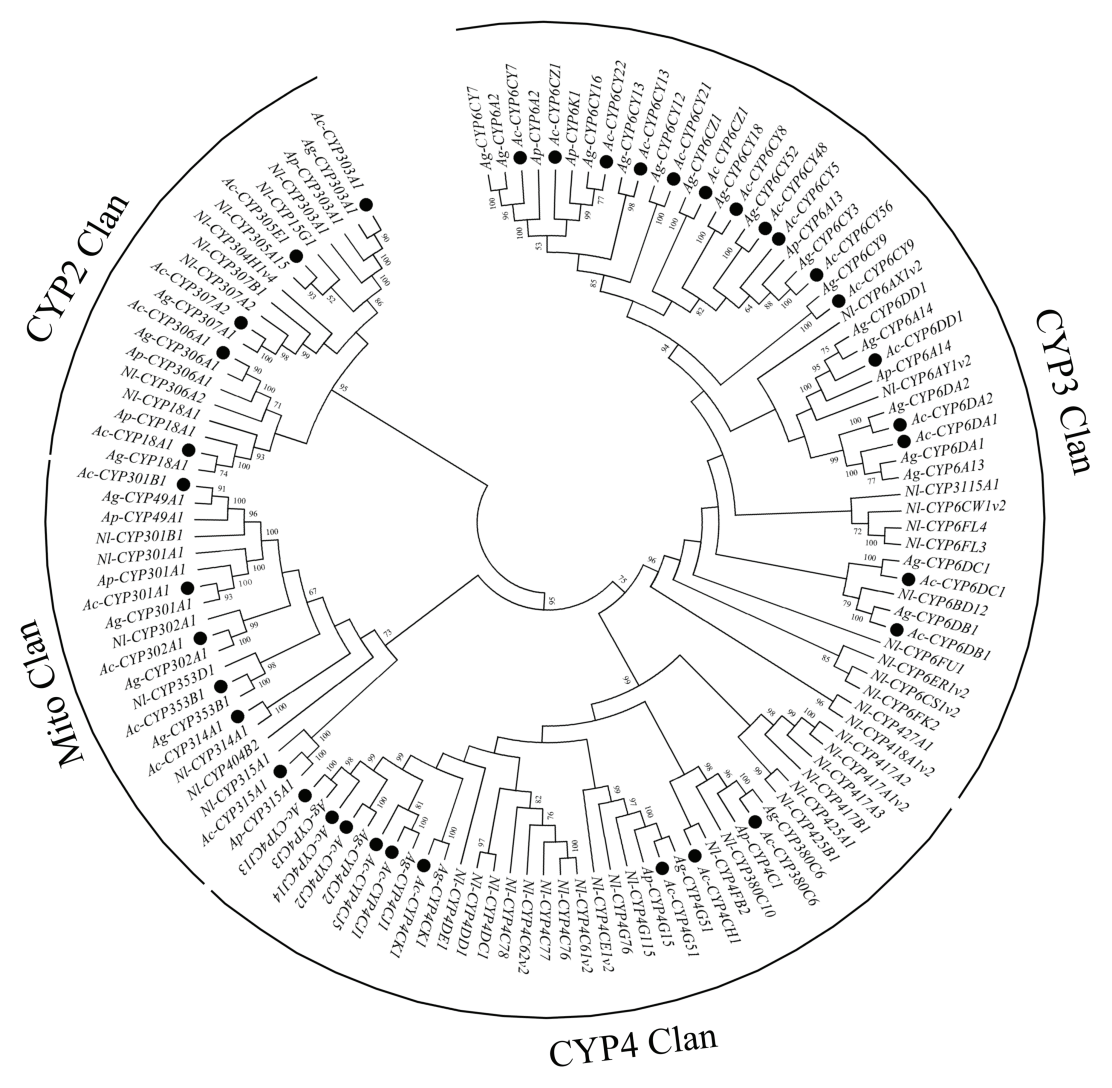
Phylogenetic analysis of P450 genes. The numbers above the branches indicate the support for the phylogenies, and only values above 50% are shown. Blackened circles indicate the *Aphis craccivora* position. Ac, *A. craccivora*; Ag, *Aphis gossypii*; Ap, *Acyrthosiphon pisum*; Nl, *Nilaparvata lugens*.

**Figure 2:**
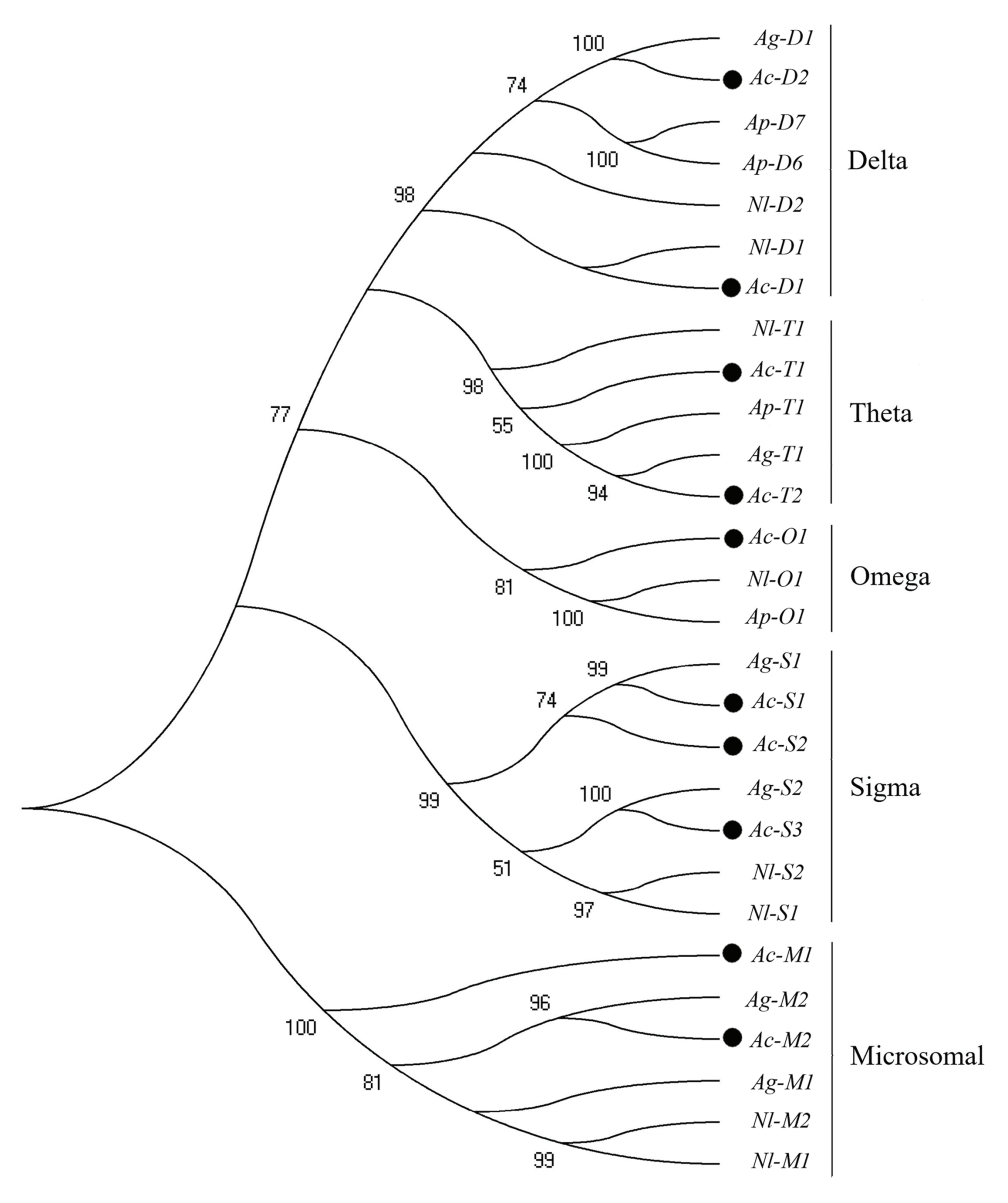
Phylogenetic analysis of GST genes. The numbers above the branches indicate the support for the phylogenies, and only values above 50% are shown. Blackened circles indicate the *A. craccivora* position. Ac, *A. craccivora*; Ag, *Aphis gossypii*; Ap, *Acyrthosiphon pisum*; Nl, *Nilaparvata lugens*.

In this study, 38 P450 genes were identified in *A. craccivora* through the transcriptomic database, including five in the CYP2 Clan, 18 in the CYP3 Clan, nine in the CYP4 Clan, and six in the Mito Clan (Figure 1). In addition, 11 P450 genes belonged to the subfamily of CYP6CY in the CYP6 family, and five P450 genes belonged to the subfamily of CYP4CJ in the CYP4 family. Ten GST genes were identified in the transcriptome of *A. craccivora*, including two Delta class GST, one Omega class GST, three Sigma class GST, two Theta class GST, and two Microsomal class GST (Figure 2).

To verify the accuracy of the transcriptome sequence, 50 genes were amplified. The electrophoretic diagram was shown in Supplementary Figure S3, and the sequence information have been submitted to the NCBI database (Supplementary Table S2).

### Expression Induction of P450 and GST Genes in the SUS Strain of *A. craccivora* by Imidacloprid

To obtain potential P450 and GST genes that might be involved in the tolerance of imidacloprid, the expression induction of all P450 and GST genes identified in the transcriptome of *A. craccivora*, was detected in the SUS strain after imidacloprid treatment with LC_15_, LC_50_, and LC_85_ doses. The amplification efficiency of each pair of primers was presented in Supplementary Table S6.

**Figure 3:**
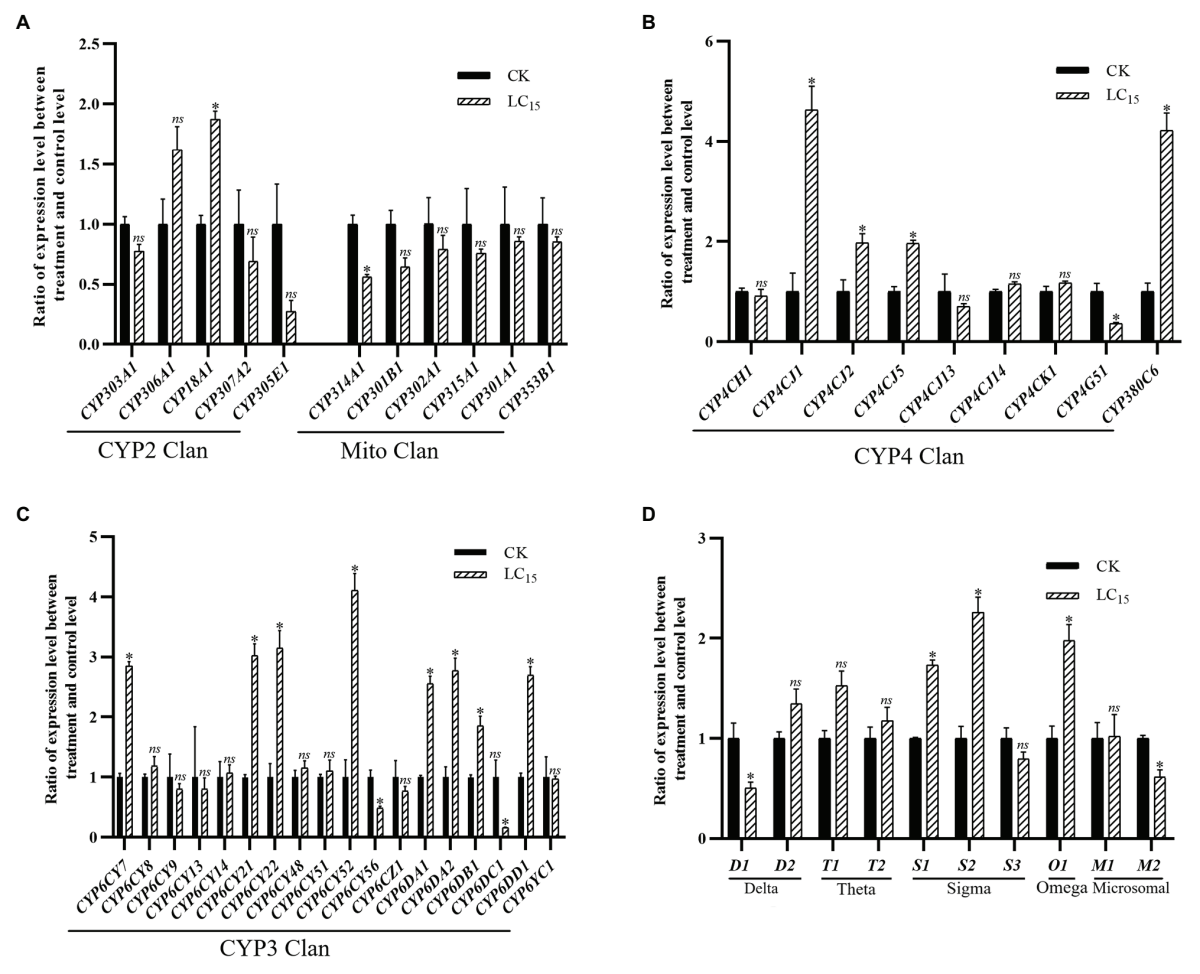
Fold changes in the expression of 38 P450 genes and 10 GST genes in the *A. craccivora* susceptible strain after the LC_15_ dose of imidacloprid treatment. The significant differences were marked by asterisks. ^*^Significantly at 0.05 level. The *ns* represents no significant difference. **(A)** Fold changes in the expression of P450 genes from CYP2 and Mito Clan. **(B)** Fold changes in the expression of P450 genes from CYP4 Clan. **(C)** Fold changes in the expression of P450 genes from CYP3 Clan. **(D)** Fold changes in the expression of GST genes.

With the LC_15_ dose of imidacloprid treatment, 22 genes were significantly up- or down-regulated in the SUS strain compared with the control (Figure 3), including 17 P450 genes (13 up-regulated, and four down-regulated) and five GST genes (three up-regulated, and two down-regulated). Among the P450 genes, one in the CYP2 Clan (Figure 3A), eight in the CYP3 Clan (Figure 3C), and four in the CYP4 Clan (Figure 3B) were up-regulated after imidacloprid treatment. In these P450 genes, the expression level of nine genes changed more than 2.0-fold, among which *CYP6CY52*, *CYP4CJ1*, and *CYP380C6* were up-regulated more than 4.0-fold. Among the GST genes, two sigma class GST genes and one omega class GST gene were up-regulated after imidacloprid treatment, and sigma two was up-regulated more than two-fold (Figure 3D).

**Figure 4:**
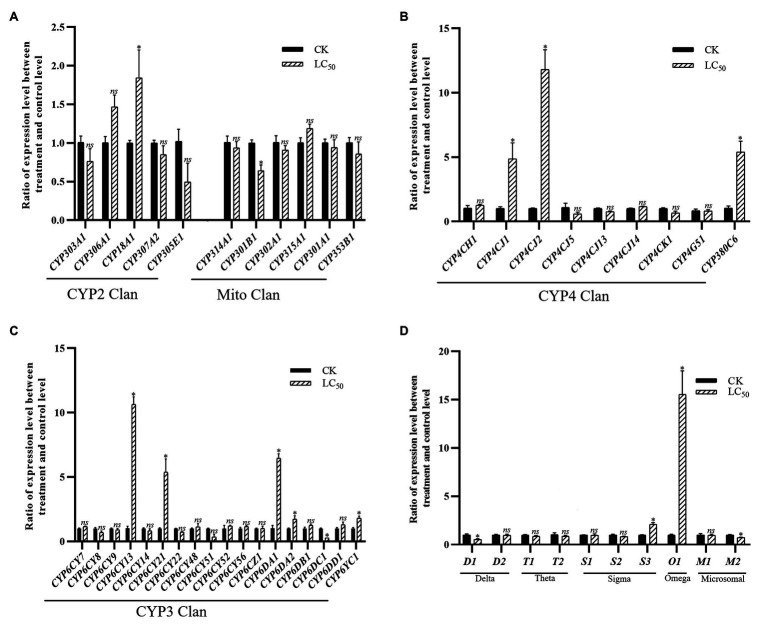
Fold changes in the expression of 38 P450 genes and 10 GST genes in the *A. craccivora* susceptible strain after the LC_50_ dose of imidacloprid treatment. The significant differences were marked by asterisks. ^*^Significantly at 0.05 level. The *ns* represents no significant difference. **(A)** Fold changes in the expression of P450 genes from CYP2 and Mito Clan. **(B)** Fold changes in the expression of P450 genes from CYP4 Clan. **(C)** Fold changes in the expression of P450 genes from CYP3 Clan. **(D)** Fold changes in the expression of GST genes.

With the LC_50_ dose of imidacloprid treatment, 15 genes were significant up- or down-regulated in the SUS strain compared with the control (Figure 4), including 11 P450 genes (nine up-regulated and two down-regulated) and four GST genes (two up-regulated and two down-regulated). Among the P450 genes, one in the CYP2 Clan (Figure 4A), five in the CYP3 Clan (Figure 4C), and three in the CYP4 Clan (Figure 4B) were up-regulated after imidacloprid treatment. In these P450 genes, the expression of six genes changed more than 2.0-fold, among which *CYP6CY13*, *CYP6CY21*, *CYP6DA1*, *CYP4CJ1*, *CYP4CJ2*, and *CYP380C6* were up-regulated more than 4.0-fold. Among the GST genes, one sigma class GST gene and one omega class GST gene were up-regulated after imidacloprid treatment (Figure 4D), and the two were up-regulated more than 2-fold.

**Figure 5:**
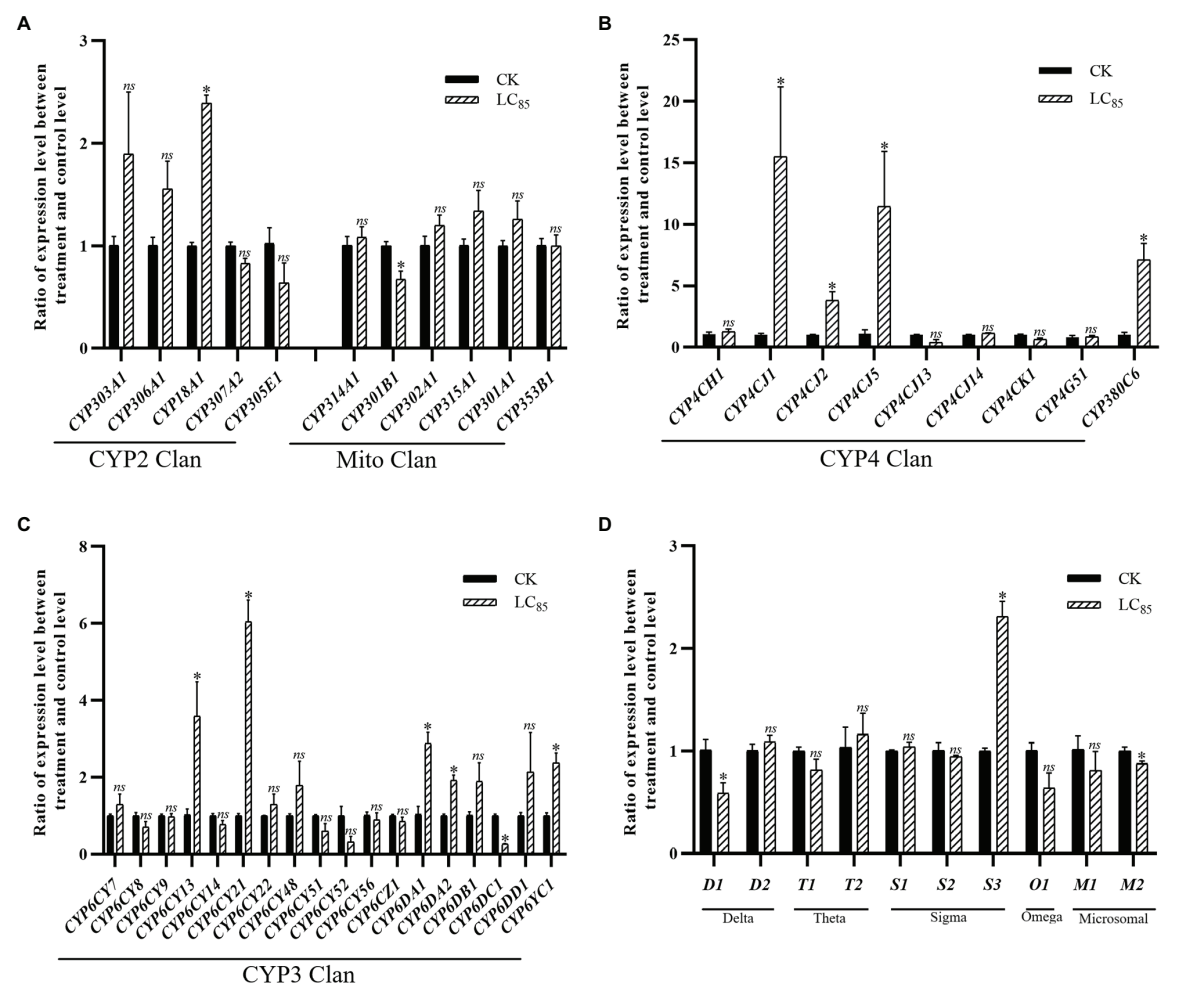
Fold changes in the expression of 38 P450 genes and 10 GST genes in the *A. craccivora* susceptible strain after the LC_85_ dose of imidacloprid treatment. The significant differences were marked by asterisks. ^*^Significantly at 0.05 level. The *ns* represents no significant difference. **(A)** Fold changes in the expression of P450 genes from CYP2 and Mito Clan. **(B)** Fold changes in the expression of P450 genes from CYP4 Clan. **(C)** Fold changes in the expression of P450 genes from CYP3 Clan. **(D)** Fold changes in the expression of GST genes.

With the LC_85_ dose of imidacloprid treatment, 15 genes were significant up-or down-regulated in the SUS strain compared with the control (Figure 5), including 12 P450 genes (10 up-regulated, and two down-regulated) and three GST genes (one up-regulated, and two down-regulated). Among the P450 genes, one in the CYP2 Clan (Figure 5A), five in the CYP3 Clan (Figure 5C), and four in the CYP4 Clan (Figure 5B) were up-regulated after imidacloprid treatment. In these P450 genes, the expression of nine genes changed more than 2.0-fold, among which *CYP6CY21*, *CYP4CJ1*, *CYP4CJ5*, and *CYP380C6* were up-regulated more than 4.0-fold. Among the GST genes, sigma3 was up-regulated after imidacloprid treatment (Figure 5D), and the fold change more than 2-fold.

In these three treatments, CYP3 and CYP4 Clans had a higher proportion of genes induced by imidacloprid (Figures 3–5). Among these genes, seven P450 genes (*CYP18A1*, *CYP6CY21*, *CYP6DA1*, *CYP6DA2*, *CYP4CJ1*, *CYP4CJ2*, and *CYP380C6*) were up-regulated in three doses of imidacloprid treatment.

### Expressive Abundance of P450 and GST Genes in the LQ and JY Populations of *A. craccivora*

To explore more potential stably over-expressing P450 and GST genes involved in the imidacloprid tolerance, the expression levels of all P450 and GST genes were detected in the *A. craccivora* populations of LQ and JY.

**Figure 6:**
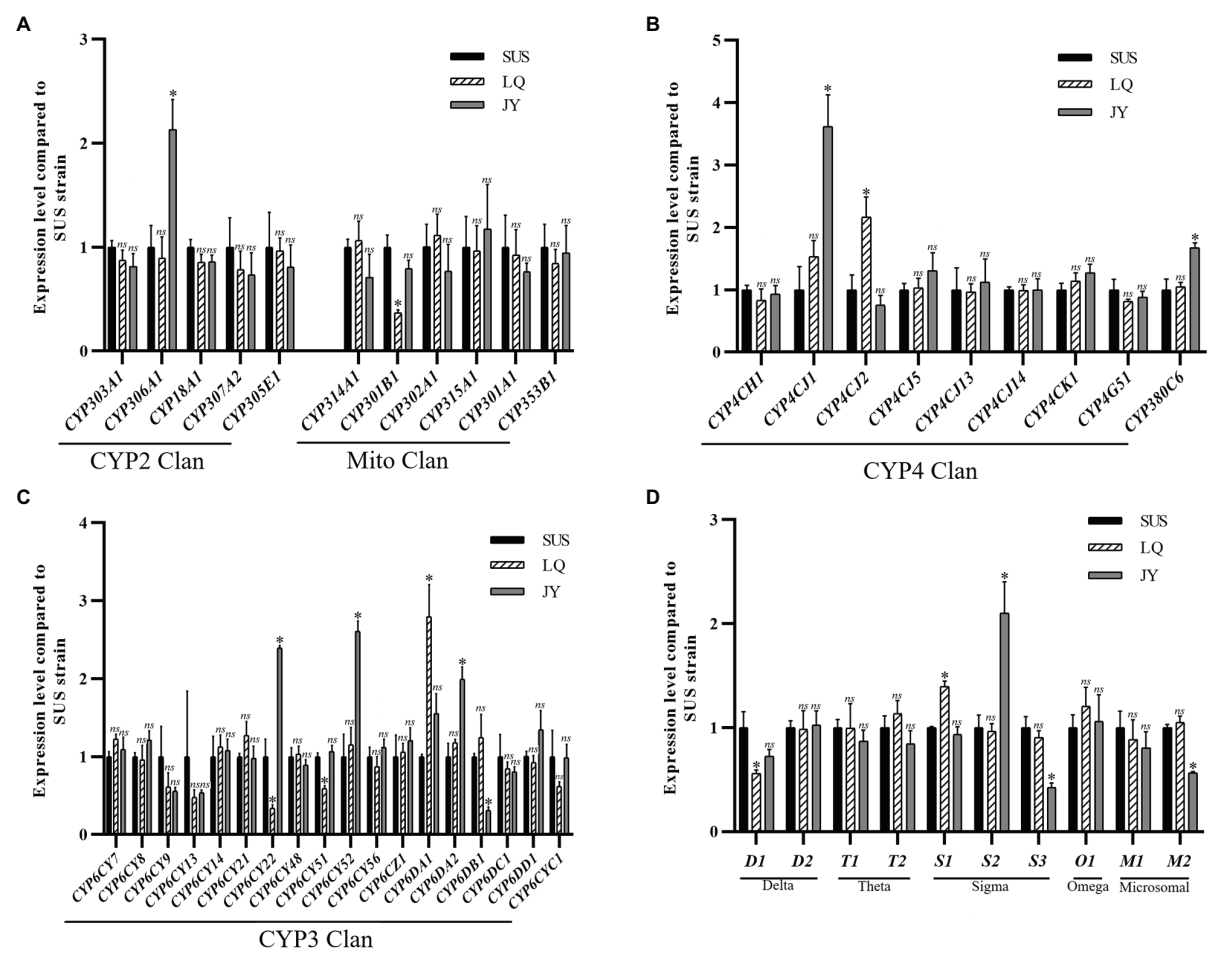
Fold changes of LQ and JY vs. SUS in the expression levels of 10 GST genes the in *A. craccivora*. The significant differences were marked by asterisks. ^*^Significantly at 0.05 level. The *ns* represents no significant difference. **(A)** Fold changes in the expression of P450 genes from CYP2 and Mito Clan. **(B)** Fold changes in the expression of P450 genes from CYP4 Clan. **(C)** Fold changes in the expression of P450 genes from CYP3 Clan. **(D)** Fold changes in the expression of GST genes.

Compared to the SUS strain, 10 genes were significantly up- or down regulated in the JY population (Figure 6), including seven P450 genes (six up-regulated and one down-regulated) and three GST genes (one up-regulated and two down-regulated). Among the P450 genes, one (*CYP306A1*) in the CYP2 Clan (Figure 6A), three (*CYP6DA2*, *CYP6CY52*, and *CYP6CY22*) in the CYP3 Clan (Figure 6C), and two (*CYP4CJ1*, *CYP380C6*) in the CYP4 Clan (Figure 6B) were up-regulated in the JY population. Except for *CYP380C6*, the fold change of the other five P450 genes were exceeded 2.0-fold. Among the GST genes, sigma3 was up-regulated in the JY population (Figure 6D), and the fold change was more than 2-fold.

Compared to the SUS strain, seven genes were significantly up- or down-regulated in the LQ population (Figure 6), including five P450 genes (two up-regulated and three down-regulated) and two GST genes (one up-regulated and one down-regulated). Among the P450 genes, one (*CYP6DA1*) in the CYP3 Clan (Figure 6C), and one (*CYP4CJ2*) in the CYP4 Clan (Figure 6B) were up-regulated in the LQ population. And the fold change of the two P450 genes were exceeded 2.0-fold. Among the GST genes, sigma1 was up-regulated in the JY population (Figure 6D).

The expression levels of *CYP6DA2*, *CYP4CJ1*, and *CYP380C6* were up-regulated after imidacloprid treatment at three doses in the SUS strain and up-regulated in the field population of JY. The results indicated that *CYP6DA2*, *CYP4CJ1*, and *CYP380C6* might play important roles in the imidacloprid tolerance in *A. craccivora*.

### RNA Interference and Effects on Insecticide Sensitivity

**Figure 7:**
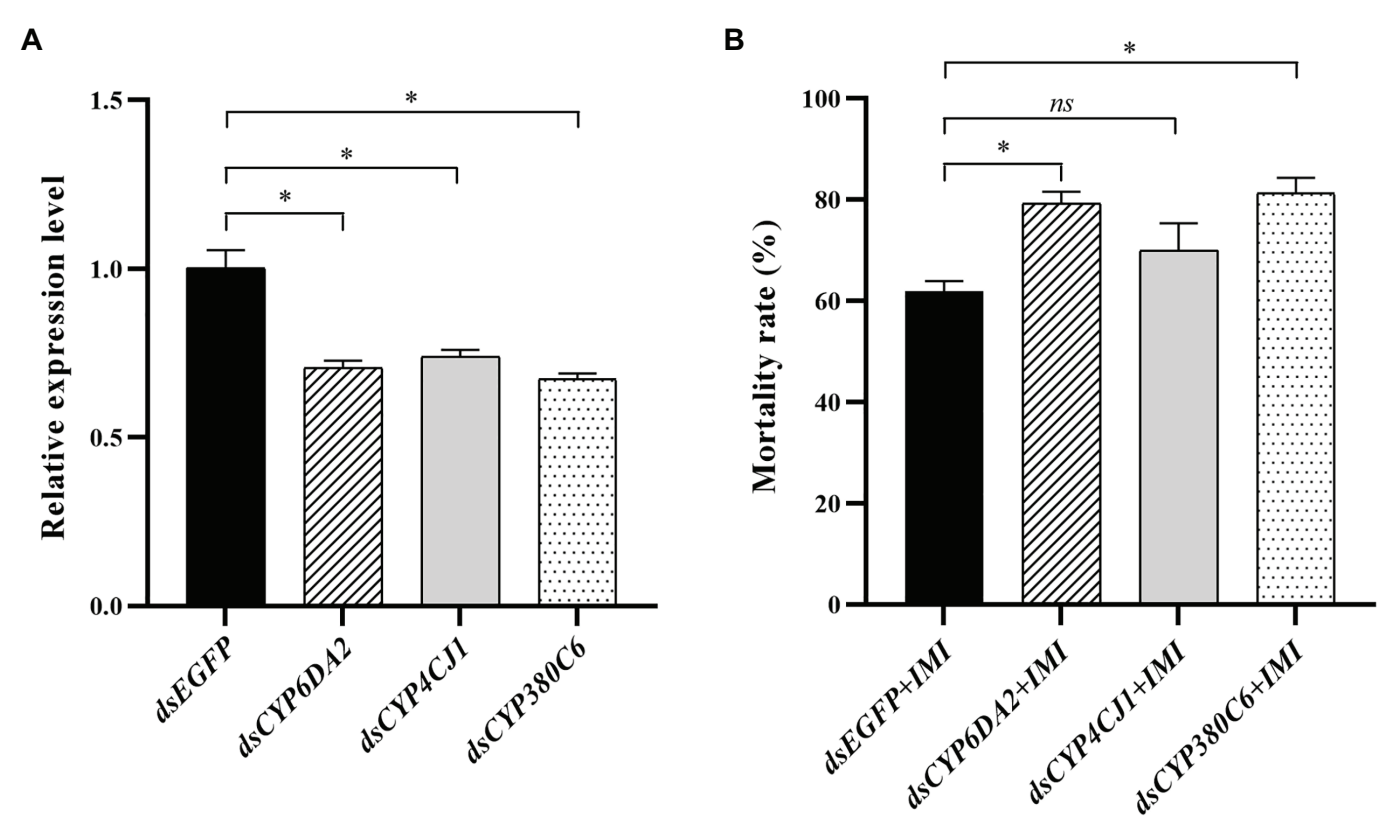
The dsRNA-mediated suppression of P450 genes and effects on imidacloprid toxicity in *A. craccivora* SUS strain. **(A)** The dsRNA-mediated suppression the expression of three P450 genes in *A. craccivora* SUS strain fed on the artificial diet with dsRNA. **(B)** The mortality of *A. craccivora* strain with LC_50_ dose of imidacloprid treatment after knockdown of *CYP6DA2*, *CYP4CJ1*, and *CYP380C6*. The significant differences were marked by asterisks. ^*^Significantly at 0.05 level. The *ns* represents no significant difference. IMI, imidacloprid.

The functions of *CYP6DA2*, *CYP4CJ1*, and *CYP380C6* in imidacloprid tolerance were analyzed by RNAi. The relative expression levels of *CYP6DA2*, *CYP4CJ1*, and *CYP380C6* were significantly decreased after dsRNA feeding in 48 h (Figure 7A). Furthermore, the mortality was increased, from 62.0% in the control to 79.3, 70.0, and 81.3% in the dsRNA-*CYP6DA2*, dsRNA-*CYP4CJ1*, and dsRNA-*CYP380C6*, with LC_50_ dose of imidacloprid treatment (Figure 7B). In addition, the mortality was significantly increased in dsRNA-*CYP6DA2* and dsRNA-*CYP380C6*, with LC_50_ dose of imidacloprid treatment (Figure 7B).

## Discussion

Imidacloprid has been extensively employed in the management of piercing-sucking pests, especially in aphids. Different levels of imidacloprid resistance have been developed in various aphids, such as *A. gossypii*, *M. persicae*, and *Aphis glycines* (Philippou et al., 2010; Yang, 2012; Cui et al., 2016). At present, imidacloprid is still used at lower frequencies in China, which may explain why the resistance of pests to imidacloprid has still increased in recent years.

In insects, metabolic resistance to insecticides is mainly due to the long-term selective pressure of exogenous compounds along with the gene amplification or overexpression of detoxification enzymes (Li et al., 2007). The detoxification enzymes, primarily P450s, GSTs, and CarEs, can metabolize insecticides to low-toxic or non-toxic substances (Enayati et al., 2005; Yan et al., 2009; Feyereisen, 2011). Transcriptome sequencing has been shown to be an efficient means of obtaining a large number of gene sequences in insect species, such as *A. gossypii* (Li et al., 2013b), and *Locusta migratoria* (Zhang et al., 2018). In the *A. craccivora* transcriptome, 38 P450 genes were identified, and most of the genes belonged to the CYP3 and CYP4 Clans, which is in keeping line with results the finding observed in other insect species (Zhang et al., 2010, 2016b; Yang and Liu, 2011). In addition, although a large number of P450 genes have been obtained in different insect species, the number of P450s in different insects varies considerably, for instance, there are 100 and 80 P450 genes in *C. quinquefasciatus* (Yang and Liu, 2011), 64 P450 genes in *Acyrthosiphon pisum* (Zhang et al., 2010), and only 36 P450 genes in *Pediculus humans* (Lee et al., 2010). In this study, only 38 P450 genes were obtained from *A. craccivora* transcriptome. Similarly, the number of P450s in *A. gossypii* obtaining from transcriptome sequencing, was only 35 (Pan et al., 2018). However, in another *A. gossypii* study, 54 P450 genes, including the sequences (<300 bp), were sequenced in transcriptome, and only 34 P450 genes in the phylogenetic analysis (Li et al., 2017). In addition, an ortholog of the juvenile hormone epoxidase, *CYP15A1*, was not identified in *A. craccivora*. Therefore, the differences of P450 gene numbers in insects may be attributable to the depth of sequencing or the insect adaptation to the environment. In insects, the CYP3 and CYP4 Clans are highly related to the development of insecticide resistance. For instance, in *Helicoverpa armigera* and *Cydia pomonella*, CYP3 and CYP4 Clans played important roles in pyrethroid insecticides, especially CYP6 and CYP9 families belonged to CYP3 Clan (Brun-Barale et al., 2010; Yang et al., 2013; Wang et al., 2019). However, in *A. craccivora*, relatively fewer P450s in CYP3 Clan lesser than in other species, were induced by insecticide. This might be related with the lack of P450 genes, or the difference of *A. craccivora’s* feeding and adaptation to the environment. However, the number of detoxification genes was few, the *A. mellifera* and *P. humans* can resist much insecticides (Claudianos et al., 2006; Lee et al., 2010).

Besides, 10 GST genes were identified in the *A. craccivora* transcriptome, including two Delta genes which play important roles in xenobiotic detoxification. In addition, the number of GST genes was relatively low in *A. craccivora*, compared with *Acyrthosiphon pisum* (Ramsey et al., 2010), *Drosophila melanogaster* (Adams et al., 2000), and *Tribolium castaneum* (Kim et al., 2010), which was 32, 40, and 41, respectively. However, only 13 protein sequences were obtained in NCBI through BLAST *A. gossypii* GST. The Delta and Epsilon classes of GSTs are insects specific and are thought to contribute to the as adaptation of insects to environmental variation (Ranson et al., 2000). In *D. melanogaster*, *C. pomonella*, and *A. pisum*, the number of The Delta and Epsilon classes were 11 and 14 (Adams et al., 2000), 4 and 5 (Ramsey et al., 2010), 10 and 0 (Hu et al., 2020). However, the number of the Delta and Epsilon classes were one and zero in *A. craccivora*. And the number of these two classes GST number were one and zero, four and zero, respectively in *A. mellifera* and *P. humans* (Claudianos et al., 2006; Lee et al., 2010). On the contrary, the number of sigma class in both fly and mosquito is only one. However, the number in *A. craccivora* is three. The *Papilio multicaudatus* has multiple sigma GSTs and are postulated to play a catalytic role, in the metabolism of plant allelochemicals (Claudianos et al., 2006). This indicated that the functional differentiation of GSTs in insect evolution and the adaptation to the environment (Hu et al., 2020).

At present, the study of insecticide resistance has changed from single gene analysis to the whole genome analysis, from detecting the expression change of a single gene to detecting the expression of multiple genes, and revealing that insecticide resistance is mediated by multiple genes (Zhu et al., 2008a,b; Feyereisen, 2011; Zhang et al., 2016a). To obtain potential P450 and GST genes that might be involved in imidacloprid resistance, all genes were detected in the SUS strain after imidacloprid treatment at three doses and detected in the field populations of JY and LQ. With imidacloprid treatment at doses of LC_15_, LC_50_, and LC_85_, 22 genes, 15 genes, and 15 genes were significantly regulated in the SUS strain compared with control, respectively. The expression levels of eight P450 genes were significantly changed under imidacloprid treatment at three doses; one (*CYP6DC1*) of them was down-regulated, while seven of them were up-regulated (*CYP18A1*, *CYP6CY21*, *CYP6DA1*, *CYP6DA2*, *CYP4CJ1*, *CYP4CJ2*, and *CYP380C6*). Six-sevenths of up-regulated P450 genes belonged to the CYP3 and CYP4 Clans, which play important roles in insecticide resistance. The over-expression of two P450 genes, *CYP6G1* and *CYP12D1*, was found to be associated with DDT resistance in *D. melanogaster* (Festucci-Buselli et al., 2005). In *M. domestica*, *CYP6A1* and *CYP6D1* have been related to pyrethroid resistance (Carino et al., 1994; Liu and Scott, 1996). The JY populations showed low level resistance to imidacloprid, and five P450 genes (*CYP6CY22*, *CYP6CY52*, *CYP6DA2*, *CYP4CJ1*, and *CYP380C6*) which belong to CYP3 and CYP4 Clans were overexpressed in the JY population compared with the SUS strain. Meanwhile, three (*CYP6DA2*, *CYP4CJ1*, and *CYP380C6*) of these genes were also up-regulated after imidacloprid treatment with three doses in the SUS strain. Genes in CYP6CY subfamily participate in neonicotinoid insecticides. In this study, *CYP6CY22* and *CYP6CY52* were up-regulated after imidacloprid treatment at three doses. In the *A. gossypii* field population, *CYP6CY22* and *CYP6CY13* were significantly up-regulated compared with the insecticide-susceptible strain. *CYP6CY22* and *CYP6CY13* can metabolize seven tested neonicotinoid insecticides (Hirata et al., 2017). Therefore, it is deduced that the two P450 genes in the CYP6CY subfamily may play important role in imidacloprid resistance. In *A. gossypii*, the expression level of *CYP4CJ1* was significantly induced by gossypol and tannic acid, and knockdown of *CYP4CJ1* could increase the sensitivity of *A. gossypii* to these two plant allelochemicals (Ma et al., 2019). Meanwhile, the expression levels of *CYP6DA2* in *A. gossypii* was significantly induced by gossypol (Peng et al., 2016). And in resistant adult *A. gossypii*, *CYP380C6* was up-regulated under spirotetramat stress (Pan et al., 2018). Besides, the expression levels of several GST genes were significantly up-regulated in the SUS strain after imidacloprid treatment at three doses. Sigma1 and Sigma2 were up-regulated after LC_15_ imidacloprid treatment. Meanwhile, Sigma3 was up-regulated after LC_50_ and LC_85_ imidacloprid treatment. Sigma class GSTs show low activity with typical GST substrates, but have high affinity for the lipid peroxidation product, 4-hydroxynonenal, and are localized in metabolically active tissues in flies (Claudianos et al., 2010). However, it is observed that these GSTs also play a role in xenobiotic detoxification and insecticide resistance (Gawande et al., 2014). *Omega1* was up-regulated after LC_15_ and LC_50_ imidacloprid treatment, and the fold change in LC_50_ was 15.5-fold. In *Leptinotarsa decemlineata*, the Omega5 was significantly overexpressed after exposure to cyhalothrin, fipronil and endosulfan (Han et al., 2016).

The *in vivo* suppression of P450 gene expression by RNAi, resulting in increased sensitivity to insecticide, has been widely used to research the contribution of P450s to insecticide resistance. In *Spodoptera frugiperda*, the second-instar larvae became more sensitive to chlorantraniliprole with a higher mortality rate than the control, after silencing *CYP321A8*, *CYP321A9*, and *CYP321B1* (Zhang et al., 2020). In *Blattella germanica*, RNAi-mediated knockdown of CYP4G19 significantly decreased its expression, which resulted in a non-uniform array of the lipid layer, enhanced cuticle permeability, and compromised insecticide tolerance (Chen et al., 2020). In *Rhynchophorus ferrugineus*, silencing of *CYP345J1* and *CYP6NR1* significantly decreased the survival rate treated with imidacloprid, indicating that overexpression of these two P450s may play an important role in developing tolerance to imidacloprid in a date palm field (Antony et al., 2019). In this study, the suppression of *CYP6DA2* and *CYP380C6* could increase the sensitivity to imidacloprid. These results indicated that, *CYP6DA2* and *CYP380C6* may contribute to resisting imidacloprid in *A. craccivora*. Meanwhile, the expression levels of *CYP6DA2* in *A. gossypii* was significantly induced by gossypol, and knockdown of *CYP6DA2* could significantly increase the toxicity of gossypol (Peng et al., 2016). Knockdown of *CYP380C6* significantly increased the sensitivity to spirotetramat in *A. gossypii* resistance strain (Pan et al., 2018). In addition, *CYP380C6* and *CYP380C9* in *M. persicae* play a crucial role in mitigating indole glucosinolate-mediated plant defense, and such effect is transgenerational (Jib et al., 2020). Therefore, it is concluded that the *CYP6DA2* and *CYP380C6* in *A. craccivora* may play important roles in insecticide tolerance and defense of plant secondary substances.

In conclusion, these results may establish a foundation for further research exploring the metabolic mechanism underlying imidacloprid resistance in *A. craccivora*. Further research may attempt to select a resistant strain in the laboratory, and perform functional expression of important genes to study the insecticide resistance in *A. craccivora*.

## Data Availability Statement

The datasets presented in this study can be found in online repositories. The names of the repository/repositories and accession number(s) can be found in the article/Supplementary Material.

## Author Contributions

Y-XY, J-HZ, and MZ conceived the study. Y-XY, R-HL, ZL, A-YW, CX, and A-LD conducted the experiments. Y-XY and R-HL analyzed the data. All authors contributed to the article and approved the submitted version.

### Conflict of Interest

The authors declare that the research was conducted in the absence of any commercial or financial relationships that could be construed as a potential conflict of interest.

## Footnotes

^1^http://www.bioinformatics.babraham.ac.uk/projects/fastqc/

^2^http://www.ncbi.nlm.nih.gov/

^3^http://www.geneontology.org

^4^http://www.expasy.ch/sprot/

^5^http://www.genome.jp/kegg/

^6^http://eggnogdb.embl.de/

^7^http://www.ncbi.nlm.nih.gov/BLAST

## Supplementary Material

Supplementary Figure S1 Nr homologous species distribution in *A. craccivora*.

Supplementary Figure S2 Gene Ontology (GO) annotation and classification of the *A. craccivora*.

Supplementary Figure S3 Electrophoretic images of 50 genes. M: marker, the marker from top to bottom are 2,000 bp, 1,500 bp, 1,000 bp, 750 bp, 250 bp, and 100 pp. 1, *CYP301B1*; 2, *CYP315A1*; 3, *CYP353B1*; 4, *CYP6CY22*; 5, *CYP6YC1*; 6, *CYP18A1*; 7, *CYP6DB1*; 8, *CYP306A1*; 9, *CYP6CY22*; 10, *CYP6CY7*; 11, *CYP4CJ2*; 12, *CYP6CY52*; 13, *CYP380C6*; 14, *CYP4CJ1*; 15, *GSTM1*; 16, *GSTD1*; 17, *CYP6CY21*; 18, *CYP6CY8*; 19, *CYP6CY5*; 20, *CYP6CY48*; 21, *CYP4CJ14*; 22, *CYP4CJ5*; 23, *CYP6DA1*; 24, *CYP6DA2*; 25, *CYP6CY14*; 26, *CYP6CZ1*; 27, *CYP314A1*; 28, *CYP6DC1*; 29, *CYP301A1*; 30, *CYP305E1*; 31, *CYP4G51*; 32, *CYP6CY13*; 33, *CYP307A2*; 34, *CYP302A1*; 35, *CYP6DD1*; 36, *CYP303A1*; 37, *CYP4CH1*; 38, *CYP4CK1*; 39, *CYP4CJ13*; 40, *CYP6CY56*; 41, *RPL11*; 42, *RPS8*; 43, *GSTM2*; 44, *CYPGSTD2*; 45, *GSTS1*; 46, *GSTS3*; 47, *GSTS2*; 48, *GSTT2*; 49, *GSTO1*; 50, *GSTT1*.
