# Supplementary material for: Function Analysis of P450 and GST Genes to Imidacloprid in Aphis craccivora (Koch)
Source: Front Physiol. 2021 Jan 20;11:624287. doi: 10.3389/fphys.2020.624287 (PMC7854575; doi:10.3389/fphys.2020.624287)
Supplement: Supplementary file 1 [file Table_1.docx]

**Table S1** The information of insect species used in phylogenetic analysis.

| Species | Gene name | Accession number | Length of amino acid |
| --- | --- | --- | --- |
| *Aphis gossypii* | CYP6CY7 | QIU80486.1 | 503 |
| *Aphis gossypii* | CYP6A2 | AML23848.1 | 517 |
| *Aphis gossypii* | CYP6CY16 | ATN96005.1 | 513 |
| *Aphis gossypii* | CYP6CY13 | QIU80470.1 | 513 |
| *Aphis gossypii* | CYP6CY12 | ATN95995.1 | 511 |
| *Aphis gossypii* | CYP6CY18 | ATN95983.1 | 514 |
| *Aphis gossypii* | CYP6CY5 | ATN95993.1 | 509 |
| *Aphis gossypii* | CYP6CY3 | ALC76824.1 | 421 |
| *Aphis gossypii* | CYP6CZ1 | ATN96010.1 | 511 |
| *Aphis gossypii* | CYP6CY9 | ATN96007.1 | 522 |
| *Aphis gossypii* | CYP6DD1 | ATN96009.1 | 512 |
| *Aphis gossypii* | CYP6A14 | XP_027842428.1 | 517 |
| *Aphis gossypii* | CYP6DA2 | ATN95992.1 | 512 |
| *Aphis gossypii* | CYP6DA1 | ATB55047.1 | 510 |
| *Aphis gossypii* | CYP6A13 | XP_027849945.1 | 519 |
| *Aphis gossypii* | CYP6DC1 | ATN96001.1 | 521 |
| *Aphis gossypii* | CYP6DB1 | ATN95990.1 | 513 |
| *Aphis gossypii* | CYP4CK1 | ATN96011.1 | 508 |
| *Aphis gossypii* | CYP380C6 | ATN95989.1 | 546 |
| *Aphis gossypii* | CYP4G51 | ATN95994.1 | 566 |
| *Aphis gossypii* | CYP4CJ1 | ATN96006.1 | 526 |
| *Aphis gossypii* | CYP4CJ2 | ATN95991.1 | 509 |
| *Aphis gossypii* | CYP4CJ3 | ATN95997.1 | 511 |
| *Aphis gossypii* | CYP353B1 | ATN95999.1 | 418 |
| *Aphis gossypii* | CYP302A1 | XP_027853688.1 | 499 |
| *Aphis gossypii* | CYP301A1 | ATN96002.1 | 525 |
| *Aphis gossypii* | CYP49A1 | XP_027845570.1 | 517 |
| *Aphis gossypii* | CYP306A1 | XP_027853760.1 | 488 |
| *Aphis gossypii* | CYP18A1 | ATN96000.1 | 512 |
| *Aphis gossypii* | CYP307A1 | XP_027848023.1 | 527 |
| *Aphis gossypii* | CYP303A1 | XP_027844048.1 | 499 |
| *Acyrthosiphon pisum* | CYP6A2 | XP_001947920.1 | 519 |
| *Acyrthosiphon pisum* | CYP6k1 | XP_001948421.1 | 514 |
| *Acyrthosiphon pisum* | CYP6A13 | NP_001353126.1 | 512 |
| *Acyrthosiphon pisum* | CYP6A14 | XP_029344865.1 | 512 |
| *Acyrthosiphon pisum* | CYP4C1 | XP_029342720.1 | 532 |
| *Acyrthosiphon pisum* | CYP4G15 | NP_001352059.1 | 517 |
| *Acyrthosiphon pisum* | CYP315A1 | XP_001944183.2 | 477 |
| *Acyrthosiphon pisum* | CYP301A1 | XP_001948959.2 | 545 |
| *Acyrthosiphon pisum* | CYP49A1 | XP_001946744.2 | 517 |
| *Acyrthosiphon pisum* | CYP306A1 | XP_029346824.1 | 492 |
| *Acyrthosiphon pisum* | CYP18A1 | XP_001947923.1 | 512 |
| *Acyrthosiphon pisum* | CYP303A1 | XP_001951093.1 | 500 |
| *Nilaparvata lugens* | CYP6AX1v2 | AIW79981.1 | 514 |
| *Nilaparvata lugens* | CYP3115A1 | AIW79991.1 | 476 |
| *Nilaparvata lugens* | CYP6CW1v2 | AIW79985.1 | 540 |
| *Nilaparvata lugens* | CYP6FL4 | AIW79989.1 | 532 |
| *Nilaparvata lugens* | CYP6FL3 | AIW79990.1 | 530 |
| *Nilaparvata lugens* | CYP6AY1v2 | QCI55729.1 | 507 |
| *Nilaparvata lugens* | CYP6FK2 | AIW79987.1 | 514 |
| *Nilaparvata lugens* | CYP6CS1v2 | AIW79984.1 | 512 |
| *Nilaparvata lugens* | CYP6ER1v2 | AUT13965.1 | 507 |
| *Nilaparvata lugens* | CYP6BD12 | AIW79983.1 | 502 |
| *Nilaparvata lugens* | CYP6FU1 | AIW79992.1 | 509 |
| *Nilaparvata lugens* | CYP427A1 | AIW79993.1 | 503 |
| *Nilaparvata lugens* | CYP418A1v2 | AIW79994.1 | 500 |
| *Nilaparvata lugens* | CYP417A2 | AIW79995.1 | 503 |
| *Nilaparvata lugens* | CYP417A1v2 | AEK01115.1 | 486 |
| *Nilaparvata lugens* | CYP417A3 | AIW80012.1 | 461 |
| *Nilaparvata lugens* | CYP417B1 | AIW80015.1 | 515 |
| *Nilaparvata lugens* | CYP425A1 | AIW80018.1 | 513 |
| *Nilaparvata lugens* | CYP425B1 | AIW80020.1 | 522 |
| *Nilaparvata lugens* | CYP380C10 | AIW79998.1 | 547 |
| *Nilaparvata lugens* | CYP4FB2 | AIW80011.1 | 509 |
| *Nilaparvata lugens* | CYP4C61v2 | AIW80000.1 | 505 |
| *Nilaparvata lugens* | CYP4C76 | AIW80001.1 | 512 |
| *Nilaparvata lugens* | CYP4C62v2 | AIW80002.1 | 520 |
| *Nilaparvata lugens* | CYP4C77 | AIW79997.1 | 509 |
| *Nilaparvata lugens* | CYP4C78 | AIW79999.1 | 589 |
| *Nilaparvata lugens* | CYP4DC1 | AIW80006.1 | 502 |
| *Nilaparvata lugens* | CYP4DD1 | AIW80007.1 | 523 |
| *Nilaparvata lugens* | CYP4CE1v2 | AIW80003.1 | 541 |
| *Nilaparvata lugens* | CYP4G115 | AIW80009.1 | 578 |
| *Nilaparvata lugens* | CYP4G76 | AIW80008.1 | 572 |
| *Nilaparvata lugens* | CYP4DE1 | QCI55730.1 | 534 |
| *Nilaparvata lugens* | CYP314A1 | APA21471.1 | 542 |
| *Nilaparvata lugens* | CYP404B2 | AIW79964.1 | 481 |
| *Nilaparvata lugens* | CYP315A1 | AIW79957.1 | 563 |
| *Nilaparvata lugens* | CYP353D1 | QCI55727.1 | 427 |
| *Nilaparvata lugens* | CYP302A1 | AIW79958.1 | 563 |
| *Nilaparvata lugens* | CYP301A1 | AIW79955.1 | 521 |
| *Nilaparvata lugens* | CYP301B1 | AIW79956.1 | 532 |
| *Nilaparvata lugens* | CYP306A2 | AIW79976.1 | 499 |
| *Nilaparvata lugens* | CYP18A1 | AIW79970.1 | 532 |
| *Nilaparvata lugens* | CYP307A2 | AIW79977.1 | 520 |
| *Nilaparvata lugens* | CYP307B1 | AIW79978.1 | 507 |
| *Nilaparvata lugens* | CYP304H1v4 | AIW79972.1 | 506 |
| *Nilaparvata lugens* | CYP305A15 | AIW79974.1 | 494 |
| *Nilaparvata lugens* | CYP15G1 | AIW79967.1 | 495 |
| *Nilaparvata lugens* | CYP303A1 | XP_022197936.1 | 755 |
| *Aphis gossypii* | GSTD1 | AFM78644.1 | 241 |
| *Aphis gossypii* | GSTT1 | XP_027838411.1 | 233 |
| *Aphis gossypii* | GSTS1 | AFM78642.1 | 203 |
| *Aphis gossypii* | GSTS2 | AFM78643.1 | 203 |
| *Aphis gossypii* | GSTM1 | XP_027839180.1 | 157 |
| *Aphis gossypii* | GSTM2 | NP_001156173.1 | 148 |
| *Acyrthosiphon pisum* | GSTD7 | AFM57708.1 | 229 |
| *Acyrthosiphon pisum* | GSTD6 | AFM57707.1 | 251 |
| *Acyrthosiphon pisum* | GSTT1 | NP_001156289.1 | 232 |
| *Acyrthosiphon pisum* | GSTO1 | NP_001155757.1 | 239 |
| *Nilaparvata lugens* | GSTD2 | AFJ75802.1 | 216 |
| *Nilaparvata lugens* | GSTT1 | AFJ75807.1 | 236 |
| *Nilaparvata lugens* | GSTO1 | AFJ75806.1 | 239 |
| *Nilaparvata lugens* | GSTD1 | AFJ75804.1 | 220 |
| *Nilaparvata lugens* | GSTS2 | AFJ75809.1 | 202 |
| *Nilaparvata lugens* | GSTS1 | AFJ75803.1 | 202 |
| *Nilaparvata lugens* | GSTM2 | AFJ75808.1 | 150 |
| *Nilaparvata lugens* | GSTM1 | AFJ75810.1 | 148 |

**Table S2** The information of specific primers used in this study.

| Gene name | Accession number | Primers in sequence verification | Primers in qRT-PCR | Amplification efficiency (%) | Primers in RNAi |
| --- | --- | --- | --- | --- | --- |
| CYP303A1 | MT130865 | F: ATGAAAAATATTCGTCAGTTTCAAA | F: GTCTTGCGGAATCGTATGGACCAA | 105.6 | - |
|  |  | R: TTATGGCGTAGGCCTCTCAG | R: CCCTGTGGTCGTCCATCAAATTCTT |  | - |
| CYP306A1 | MT130852 | F: ATGTTTTGGATCATTGGTGTAATA | F: CTTATTGCCGACGCCGTGTCTACTA | 91.5 | - |
|  |  | R: CTAAATTCGAGATTTAAAAACGATG | R: TAGGGTCATCTTCTGGCCACGC |  | - |
| CYP18A1 | MT130854 | F: ATGACGACCGAGATCATGTCC | F: TGAAGCAGATCACCGGTCGTCC | 90.5 | - |
|  |  | R: CTATTTGTTGCGTGCTTTCATAA | R: TCCATGTTCAACCTGTTGTGGCC |  | - |
| CYP307A2 | MT130868 | F: GCGGCGTACCCGGTCAT | F: CAACGACGACGACGAACAA | 104.4 | - |
|  |  | R: CTATGGAGATCCGTCGAGCG | R: CGAATGACCGCCGATGAAG |  | - |
| CYP305E1 | MT130871 | F: CTAACCTGTCCCAGCGCTAA | F: GTGTGACGCCGACCTTATACTTGCA | 99.3 | - |
|  |  | R: GGTTTTGGTGTCATAAGTATACCACA | R: TCTGGTTAAGCAGCCCACCAGCC |  | - |
| CYP314A1 | MT130873 | F: ATGGTACTACAGGAAAAATATTGGA | F: GGCGAACTACAGAAAGTCTCGACCG | 107.3 | - |
|  |  | R: TCAACAATTTCTGTCTCGTAGAATA | R: TGTCTCTCGCTTGGGAAATCAGGT |  | - |
| CYP301B1 | MT130860 | F: ATGTCCGTGTTGGCCAAG | F: CCTATGCTGCAACCAAGAACAGCA | 108.3 | - |
|  |  | R: TTAGTCTTCAATCATTTTGAATTTCA | R: GTGTTCATCTTCAAGGCACCCGAG |  | - |
| CYP302A1 | MT130867 | F: ATGCGACGAGTCAATCATTG | F: CGGCGTGTCCATAGTGTGGATATTC | 99.4 | - |
|  |  | R: TCATTTAATAATCGTTTTAATCACG | R: ATCCTTGCCATTTGTGGGCAATAGT |  | - |
| CYP315A1 | MT130859 | F: TTACCAATAGTTGGTACAATGTTTTC | F: GGCTGCTGGTGGTGGTCGTAAA | 94.0 | - |
|  |  | R: TTTTATATTTATGTGATTATCTGGAACTG | R: CCACCATTCTTCTCCATCCATGAAAT |  | - |
| CYP301A1 | MT130856 | F: ATGTGGATACTAGTGCTAGTACTATTCAG | F: GAAATCTTGTTGGTCGACCGGATTT | 96.8 | - |
|  |  | R: CTAATTATGTATGTACTTAGGCCTATCAT | R: CCGTGTACTCCGACAACACCTGG |  | - |
| CYP353B1 | MT130858 | F: ATGACTTACCGTCTGTGCAAGC | F: CAGCGAGACCCATATTTCCAGCTTT | 94.6 | - |
|  |  | R: ATCTATCGTGGAATATTAAATCTATGC | R: CCATTGCTGCTTCTTTATGCTGAGG |  | - |
| CYP6CY7 | MT130850 | F: ATGATTGAAATTTTTTCGAGTTG | F: GGGTGCACTGAGCAACTTATGAACG | 103.6 | - |
|  |  | R: TTAATTTACAACGATCGGCTTAA | R: CTGTTTGAAGCTTGGCCGGAATATT |  | - |
| CYP6CY8 | MT130882 | F: ATGGAGTCACTTATACGAATTGTAAAT | F: TACTGTGCGGTCGCAGGATTCC | 100.3 | - |
|  |  | R: TTAAAGCTTTTCTAACTGCGTCC | R: CGCTCTGTTGCCGTGTTTCGTAC |  | - |
| CYP6CY9 | MT130857 | F: ATGTTCGCCGCGCTACAG | F: GACCGTGTTCCAACCTTCGTTCC | 107.4 | - |
|  |  | R: GTTTAAATTTTAACCAAATGCCG | R: CCTGCCGGTACCGCATAGTGTC |  | - |
| CYP6CY13 | MT130869 | F: ATGATTTCGTGGACGATCAATT | F: AACAAATACGCCACCGACGTCATAG | 90.3 | - |
|  |  | R: TTAAACCGCAGCGACTGG | R: GGTGAAATCAATTGCAACAGGTTGG |  | - |
| CYP6CY14 | MT130879 | F: ATATTACGTTTTCTCAGATTTGAATTC | F: CGGTAAACGTAATGGCGAGAAGCTT | 92.8 | - |
|  |  | R: TTAATCCACAATTGGCTTAAAATTT | R: AACTGATCGCTGCACTCTCGGATT |  | - |
| CYP6CY21 | CYP6CY21 | F: ATGATTTCGAGTGATATTATTATCAATT | F: ACTTACGTCGCCGAGATTGACAAAA | 102.0 | - |
|  |  | R: TCATTTTGAAATCGATTGAAAAC | R: CTTGCTTCTATCAAGCTCTGCGCC |  | - |
| CYP6CY22 | MT130851 | F: ATGATATCATGTCTGTCTAACTTGT | F: TACAGATGCTGGCGGTGATCTGC | 103.5 | - |
|  |  | R: TTAATTTTCAATGATCGGTCTAA | R: TTTACGCGCCTGCATAAGGGTCT |  | - |
| CYP6CY48 | CYP6CY48 | F: ATGTATCCATCGCACATCACC | F: GCTATACAGACATACCGAGTGCCCAA | 92.80 | - |
|  |  | R: CCAAATGCCATTTTTTGGTACTA | R: GTAGGTAAACACCGCTTGGCCGT |  | - |
| CYP6CY51 | MT130881 | F: ATGTATTCATCGTCCATCGCC | F: AGGACTGTTTCAGATGAGGACGCCT | 94.5 | - |
|  |  | R: ATTTTCGGAAAGTTTCAGTTCAAAC | R: CGACAATGGATTTACCGCGAAGTC |  | - |
| CYP6CY52 | MT130848 | F: TGTATTTATATTTTAACCATGATTT | F: GCACAAACATTGATGCAAGCCAGA | 95.9 | - |
|  |  | R: CTATTGTCTAATGATTGGTTTCA | R: TGTTGGATACAGGTTCAGTACCGGC |  | - |
| CYP6CY56 | MT130861 | F: TGTATTTATATTTTAACCATGATTTCG | F: ACTTTGCGCACTTCACTGACCATG | 93.7 | - |
|  |  | R: CTATTGTCTAATGATTGGTTTCAGAG | R: GTTTACCGGACGTGAATCCAGGG |  | - |
| CYP6CZ1 | MT130874 | F: CAGACGTTCGTCCGAACAGTA | F: CACATGGACCCACAGCCACTGGT | 101.4 | - |
|  |  | R: TTAAGTATTCAATTTCCTAAATTTTAACC | R: ATCGTATAAATGCCACCGAACCGTT |  | - |
| CYP6DA1 | MT130877 | F: ATGTCGGACGTGTGGCC | F: GGTATTTCGGCATCTTCGAGGGA | 106.4 | - |
|  |  | R: TTATTGATCAATACTTTTCAACGATAA | R: CGCAAGTTCACCAGGTGATCGAA |  | - |
| CYP6DA2 | MT130876 | F: ATGATCTGCTTCGGTTGTTG | F: CGTACTGGTACACTCAGTGCTGGCC | 100.9 | F: *TAATACGACTCACTATAGGG*TGCATTTATATTTTTTGCGG |
|  |  | R: TCACTTAATTCGCTTAAACAATAAAC | R: GCTTTGAGCTTGGCCACGGTAA |  | R: *TAATACGACTCACTATAGGG*CTAGTCCTGTTTTGGCCTCC |
| CYP6DB1 | MT130853 | F: ATGATTTCAAAGGAAACAATATT | F: GGTCGCCGAGCATCATTGGTTA | 105.0 | - |
|  |  | R: CTATTCACTCAACTTATTGAATGAC | R: CTCTCATTCGTTTCCATTTGGAACC |  | - |
| CYP6DC1 | MT130872 | F: ATGGTTTTATTTGATTGTTTGCTAT | F: GAGGCACCAGCAGTGATTGATGAAC | 91.4 | - |
|  |  | R: TTAGTTTTTTCTCTTGGTAACATGAA | R: AGCATTCGACCGACCTTCCGA |  | - |
| CYP6DD1 | MT130866 | F: ATGTTTCCAGCAGCCATCAT | F: TCCTCGTGAAAAGTACGTGGGCAT | 96.7 | - |
|  |  | R: TCAATGTGTACGTTTATTGACGTT | R: TCAACGGTTCTCTGTGACCGTCG |  | - |
| CYP6YC1 | MT130855 | F: CGATTTCAAACAGATTTTCAGGA | F: GCGACAAAATCGACGCAGATGTT | 107.3 | - |
|  |  | R: AACATAAATTTCTCGTTGTTGCC | R: TCCAGCTCGCTATCCAAGTGCTCT |  | - |
| CYP4CH1 | MT130864 | F: ATGTACACACTCATACAAAAGGTCATA | F: CAGCCGGTTCAACGTTGATGGT | 96.3 | - |
|  |  | R: CTACGAAGTTCTTGGTATTATTTTGAC | R: CGGTTTTCATTTGGAGCATGCC |  | - |
| CYP4CJ1 | MT130879 | F: ATGATCGTCTACAATTTGATCGG | F: GGAAACTTACGTGCCGTCACTCAAT | 92.7 | F: *TAATACGACTCACTATAGGG*TCACTCAATAATCACTCCCG |
|  |  | R: TTACGTATGGCTTGTGATACTCTTG | R: CCGTCGATTTACCCTCTTGAGTACG |  | R: *TAATACGACTCACTATAGGGG*AAAAGAAACGTATCCACTT |
| CYP4CJ2 | MT130849 | F: ATGTTGGAACCCAACCTTGTCTA | F: TGTTCTCGTGCACCATAGCGATG | 106.4 | - |
|  |  | R: CTAAAGTTTATGAAATGTTACTTTT | R: CAACATTTCATACTCGGGACCCTTG |  | - |
| CYP4CJ5 | MT130878 | F: ATGACAATTTTTTTGTTACTAAGTTTGT | F: CTGTGCTCGGAGACCCAAGGAAC | 96.4 | - |
|  |  | R: TTATTTAATCTTGGTTAATTTGATATCAG | R: TTATATGCACCAATGGCCCATGGT |  | - |
| CYP4CJ13 | MT130862 | F: ATGATCGAATTAAACGTTTACAGTG | F: CACGTGCCATCCGTAACGAAAAC | 97.7 | - |
|  |  | R: TTAAAGTTTATAAAATTTCATCTTTGGA | R: TTAGCAAAACGTATGATCGGCCTGA |  | - |
| CYP4CJ14 | MT130879 | F: ATGATCGATCTAAACGTTTACGG | AGCGCTGGTGCCAGAAATTGTATT | 95.6 | - |
|  |  | R: TTATTTAAGTTTAAAAAAATTCATCTTTG | R: TTCTCAAGATCAATTGCGCGCTAAC |  | - |
| CYP4CK1 | MT130863 | F: ATCGGTCCGGTGGTTCG | F: CCATTCATCGGTACCGGATTAGTGA | 94.2 | - |
|  |  | R: CTACGTCCGTTCTTCAAGTGTC | R: GCAGCCAATTTAGCGTAAGGCTTTA |  | - |
| CYP4G51 | MT130870 | F: ATGGCGACCAACATTCAGG | F: CGTAAGCAGCTCTATGACCTTGCCA | 103.6 | - |
|  |  | R: TTAAGCAGCGCTCTTAGCTAGTG | R: ATAACCTGGGCCCGATCCACAT |  | - |
| CYP380C6 | MT130847 | F: AGACGATTTCCTTCCTACGA | F: CCGCCGTCATACCCAATTATTGG | 106.4 | F: *TAATACGACTCACTATAGGG*TTATTAGATTTGAATGAAGC |
|  |  | R: TTAAAATATTCTTATTTTTTTTTTATATG | R: CTCCAGGGCCTTGGAACTGTTTAGT |  | R: *TAATACGACTCACTATAGGG*TAAAACTATATTTATGGCGT |
| GSTD1 | MT130836 | F: ATGACTATTGATTTCTACTATGCAAGGT | F: CAGAAGAGCATCCGATTATTTCGGC | 107.1 | - |
|  |  | R: TTAATCTAGCAGTTTTGAAAACAAATA | R: CGGCGAGAGTTACTTCAGGTCCAG |  | - |
| GSTD2 | MT130837 | F: ATGCCGATCGACTTTTACTACA | F: TCCACCACGGTGAACACATGAAAC | 90.5 | - |
|  |  | R: CTATTTCTTGGTGAGGTTAGCCA | R: AGCGCTTGCTTCTTCGGGTCTT |  | - |
| GSTT1 | MT130838 | F: ATGGTTAAATTAAAGTTTTATTACGACT | F: ACTGGCCAGGCAGCAAATGTTG | 92.4 | - |
|  |  | R: TTAACTATTCTTTTTACTTAATTCACTAGC | R: CCACTTCACATGCAGCAACAAGATC |  | - |
| GSTT2 | MT130839 | F: ATGGCAAAGTTAATATTGTATTATGAT | F: CACCCGAATTTGTAGCCCTA | 102.7 | - |
|  |  | R: TTATATTTTTGAATTCAATTTACCATA | R: TGTGTTGCCACTCAAGGT |  | - |
| GSTS1 | MT130840 | F: ATGACGTCGTACAAAGTCACTTACT | F: CGACATCACTGCTCTGGCTGAACC | 103.7 | - |
|  |  | R: TTAAGGATTATTTGTTGGTCGTTTAG | R: CAATTTCCAATGTTGGAACTTTGCC |  | - |
| GSTS2 | MT130841 | F: ATGACGTCCTATAAATTGACGTATTT | F: CTCGAGCCGAACAGATCCGATTC | 106.8 | - |
|  |  | R: TCATAGTAAACTTTTTGGTCGTTTAG | R: CCGACAAATGGCTACGGACTGATTA |  | - |
| GSTS3 | MT130842 | F: ATGTCGGTGTATAAACTCACATACTT | F: CTTCAATTTTCCTGCACTGGCAGAA | 95.3 | - |
|  |  | R: TTATAATATTATGGAGTTTGCAGGTC | R: GGAACTTTTCCAAATGGCATTGTGG |  | - |
| GSTO1 | MT130843 | F: ATGTCCACCAAACACTTTGCCGG | F: CACGCCGCATGATACAGT | 92.2 | - |
|  |  | R: AAAGTCATAAGCAGGTAATCCGG | R: GCAACAGGCTTTCAGAGAGA |  | - |
| GSTM1 | MT130844 | F: ATGAAGGTTGCATTGGAAATC | F: GGTTGCATTGGAAATCGACGAAG | 101.4 | - |
|  |  | R: TTAAACAAAACTTAAAATAACTTGAAAAG | R: TTCATTTTTGCATCTTCGGGGTTAG |  | - |
| GSTM2 | MT130845 | F: ATCAGCAAAGGAGGTGGGGAAGT | F: CGAAAGAGTAAGAAGGGCAC | 95.9 | - |
|  |  | R: AATATTATGCATACTTAAGATAA | R: CCTTGTAGGTTGGGGTAGTATG |  | - |
| RPS8 | GAJW01000269 | F: ATGGGTATATCCCGTGATACATG | F: ACTAAGCTTGGAGCTCGCCGTGTAC | 107.4 | - |
|  |  | R: TTATTTGCCTTTCTTGGCCTT | R: TCTGACCAACTCATTGTTTGATGCG |  | - |
| RPL11 | GAJW01000099 | F: ATGCCTTTCAAGAGGTTTGTTGA | F: TGGCCGAGTGGTCTACGTTGTTG | 103.6 | - |
|  |  | R: TTACGCATTAGCAACTGCTGTCT | R: TGGCTGATACGCATCTTTGACCTG |  | - |
| EGFP |  | **-** | **-** | **-** | F: *TAATACGACTCACTATAGGG*AAGTTCAGCGTGTCCG |
|  |  | - | - |  | R: *TAATACGACTCACTATAGGG*CACCTTGATGCCGTTC |

**Table S3** Summary statistics of the *A. craccivora* transcriptome.

| Statistical content | Number |
| --- | --- |
| Total raw reads | 54,778,308 |
| Total clean reads | 52,576,182 |
| Q_20_ percentage | 98.58% |
| GC percentage | 48.74% |
| transcripts | 39,048 |
| Total length of transcripts | 42,850,961 |
| Mean length of transcripts | 689 |
| N50 of transcripts | 1,852 |
| Unigenes | 22,894 |
| Total length of Unigenes | 22,171,709 |
| Mean length of Unigenes | 497 |
| N50 of Unigenes | 1,790 |

**Table S4** The BLAST results of P450 and GST genes.

| Gene name | Accession number | ORF (bp) | BLAST species | BLAST name | Accession number | Score | E-value |
| --- | --- | --- | --- | --- | --- | --- | --- |
| CYP303A1 | MT130865 | 1074 | *Aphis gossypii* | CYP303A1 | XP_027844048.1 | 726 | 0.00 |
| CYP306A1 | MT130852 | 1479 | *Aphis gossypii* | CYP306A1 | ATN95988.1 | 1002 | 0.00 |
| CYP18A1 | MT130854 | 1539 | *Aphis gossypii* | CYP18A1 | XP_027853767.1 | 1069 | 0.00 |
| CYP307A2 | MT130868 | 1443 | *Aphis gossypii* | CYP307A2 | QIU80466.1 | 976 | 0.00 |
| CYP305E1 | MT130871 | 1506 | *Aphis gossypii* | CYP305A1 | ATB55046.1 | 984 | 0.00 |
| CYP314A1 | MT130873 | 1560 | *Aphis citricidus* | CYP314A1 | ARW79963.1 | 1051 | 0.00 |
| CYP301B1 | MT130860 | 1554 | *Aphis gossypii* | CYP49A1 | ATN96003.1 | 1071 | 0.00 |
| CYP302A1 | MT130867 | 990 | *Aphis gossypii* | CYP302A1 | ATN95985.1 | 638 | 0.00 |
| CYP315A1 | MT130859 | 1314 | *Aphis citricidus* | CYP315A1 | ARW79962.1 | 880 | 0.00 |
| CYP301A1 | MT130856 | 1578 | *Aphis gossypii* | CYP301B1 | QIU80472.1 | 1081 | 0.00 |
| CYP353B1 | MT130858 | 1356 | *Rhopalosiphum maidis* | CYP49A1 | XP_026816007.1 | 872 | 0.00 |
| CYP6CY7 | MT130850 | 1554 | *Aphis glycines* | CYP6CY7 | QEE13702.1 | 977 | 0.00 |
| CYP6CY8 | MT130882 | 1998 | *Aphis gossypii* | CYPCY18 | ATN95983.1 | 664 | 0.00 |
| CYP6CY9 | MT130857 | 1560 | *Aphis gossypii* | CYP6CY9 | QIU80484.1 | 1001 | 0.00 |
| CYP6CY13 | MT130869 | 1542 | *Aphis gossypii* | CYPCY13 | QIU80470.1 | 999 | 0.00 |
| CYP6CY14 | MT130879 | 1650 | *Aphis pomi* | CYP6CY14 | QBP33516.1 | 1051 | 0.00 |
| CYP6CY21 | CYP6CY21 | 1539 | *Aphis gossypii* | CYP6CY13 | QIU80505.1 | 1030 | 0.00 |
| CYP6CY22 | MT130851 | 1542 | *Aphis gossypii* | CYP6CY22 | ATB55049.1 | 986 | 0.00 |
| CYP6CY48 | CYP6CY48 | 1545 | *Aphis gossypii* | CYP6CY5 | ATN95993.1 | 996 | 0.00 |
| CYP6CY51 | MT130881 | 1059 | *Rhopalosiphum padi* | CYP6CY3-3 | QJA10287.1 | 597 | 0.00 |
| CYP6CY52 | MT130848 | 1560 | *Aphis gossypii* | CYPCY19 | QIU80479.1 | 958 | 0.00 |
| CYP6CY56 | MT130861 | 1560 | *Aphis glycines* | CYP6CY20 | AXJ21603.1 | 907 | 0.00 |
| CYP6CZ1 | MT130874 | 1596 | *Aphis pomi* | CYP6CY14 | QBP33516.1 | 1032 | 0.00 |
| CYP6DA1 | MT130877 | 1539 | *Aphis gossypii* | CYP6A13 | AML23849.1 | 1021 | 0.00 |
| CYP6DA2 | MT130876 | 1539 | *Aphis gossypii* | CYP6DA2 | ATN95992.1 | 1003 | 0.00 |
| CYP6DB1 | MT130853 | 1542 | *Aphis gossypii* | CYP6K1-like | XP_027848748.1 | 1062 | 0.00 |
| CYP6DC1 | MT130872 | 1566 | *Aphis gossypii* | CYP6J1-like | XP_027852586.1 | 1061 | 0.00 |
| CYP6DD1 | MT130866 | 1536 | *Aphis glycines* | CYP6DD1 | QEE13699.1 | 1035 | 0.00 |
| CYP6YC1 | MT130855 | 888 | *Aphis gossypii* | CYP6UN1 | ATN95984.1 | 599 | 0.00 |
| CYP4CH1 | MT130864 | 1575 | *Aphis gossypii* | CYP4CH1 | QIU80488.1 | 1053 | 0.00 |
| CYP4CJ1 | MT130879 | 1581 | *Aphis gossypii* | CYP4CJ1 | QIU80478.1 | 1061 | 0.00 |
| CYP4CJ2 | MT130849 | 1530 | *Aphis gossypii* | CYP4V2-like | XP_027840280.1 | 892 | 0.00 |
| CYP4CJ5 | MT130878 | 1530 | *Aphis glycines* | CYP4C1-like | KAE9536823.1 | 1042 | 0.00 |
| CYP4CJ13 | MT130862 | 1533 | *Aphis gossypii* | CYP4CJ13 | QIU80511.1 | 1026 | 0.00 |
| CYP4CJ14 | MT130879 | 1536 | *Aphis gossypii* | CYP4CJ14 | QIU80508.1 | 940 | 0.00 |
| CYP4CK1 | MT130863 | 1467 | *Aphis gossypii* | CYP4CK1 | ATN96011.1 | 1021 | 0.00 |
| CYP4G51 | MT130870 | 1701 | *Aphis glycines* | CYP4G51 | AXJ21604.1 | 1164 | 0.00 |
| CYP380C6 | MT130847 | 1653 | *Aphis gossypii* | CYP380C6 | QIU80502.1 | 1066 | 0.00 |
| GSTD1 | MT130836 | 720 | *Daktulosphaira vitifoliae* | GSTD1 | AUN35382.1 | 316 | 3e-106 |
| GSTD2 | MT130837 | 648 | *Aphis gossypii* | GSTD1 | AFM78644.1 | 490 | 4e-175 |
| GSTT1 | MT130838 | 663 | *Aphis gossypii* | GSTT1-like | XP_027838411.1 | 468 | 3e-166 |
| GSTT2 | MT130839 | 699 | *Aphis gossypii* | GSTT1-like X1 | XP_027838412.1 | 458 | 1e-162 |
| GSTS1 | MT130840 | 612 | *Melanaphis sacchari* | GSTS1 | AWL24830.1 | 203 | 2e-136 |
| GSTS2 | MT130841 | 612 | *Aphis gossypii* | GSTS2 | AFM78643.1 | 404 | 3e-142 |
| GSTS3 | MT130842 | 618 | *Rhopalosiphum padi* | GSTS1 | XP_026821054.1 | 370 | 8e-12 |
| GSTO1 | MT130843 | 717 | *Acyrthosiphon pisum* | GSTO1-like | NP_001155757.1 | 402 | 5e-140 |
| GSTM1 | MT130844 | 447 | *Aphis gossypii* | GSTM1-like | XP_027846548.1 | 295 | 1e-100 |
| GSTM2 | MT130845 | 471 | *Aphis gossypii* | GSTM1-like | XP_027839180.1 | 303 | 2e-103 |
