# Supplementary figures and images for: Function Analysis of P450 and GST Genes to Imidacloprid in Aphis craccivora (Koch)
Source: Front Physiol. 2021 Jan 20;11:624287. doi: 10.3389/fphys.2020.624287 (PMC7854575; doi:10.3389/fphys.2020.624287)

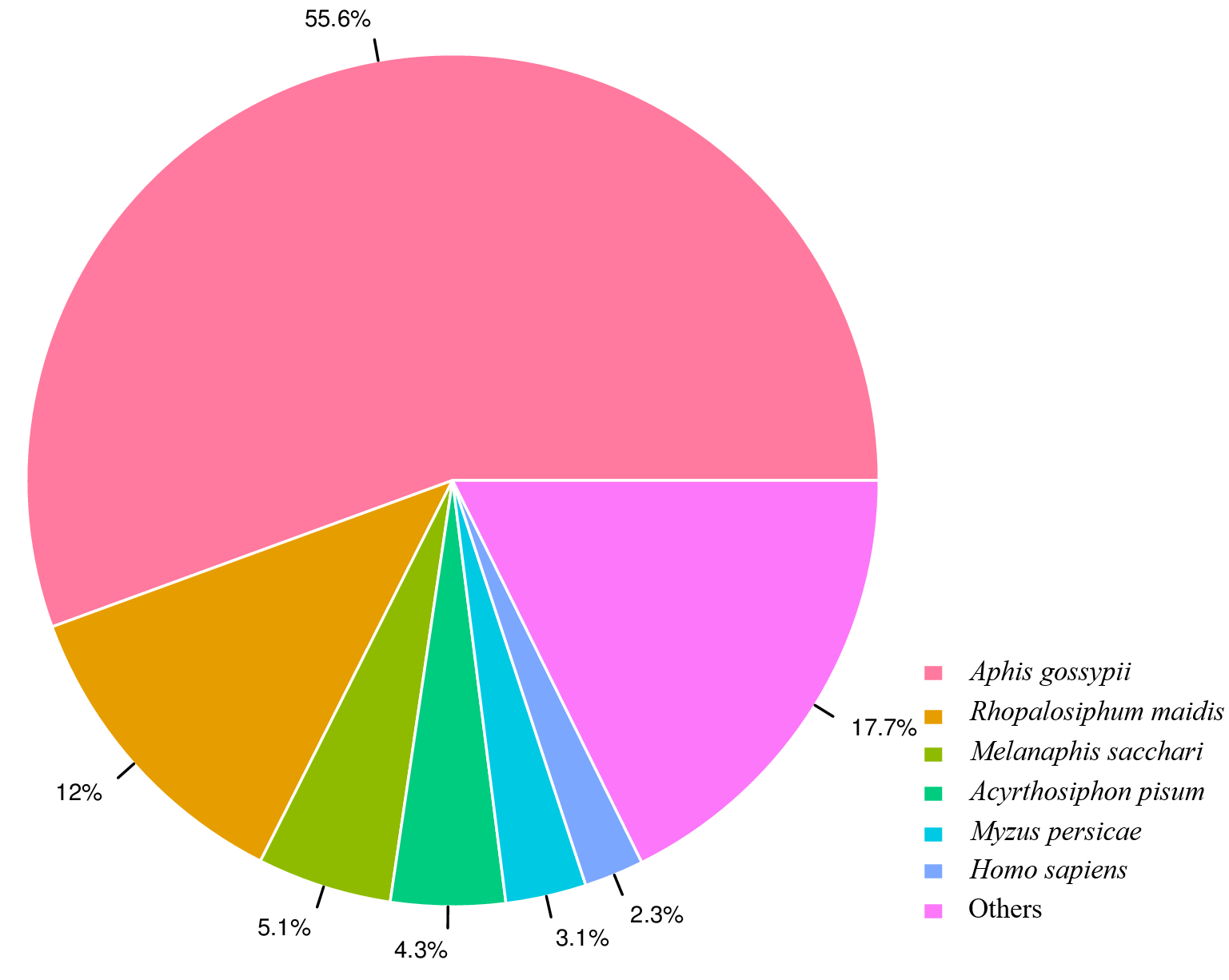

Supplement: Supplementary Figure S1 — Nr homologous species distribution in A. craccivora. [file Image_1.tiff]

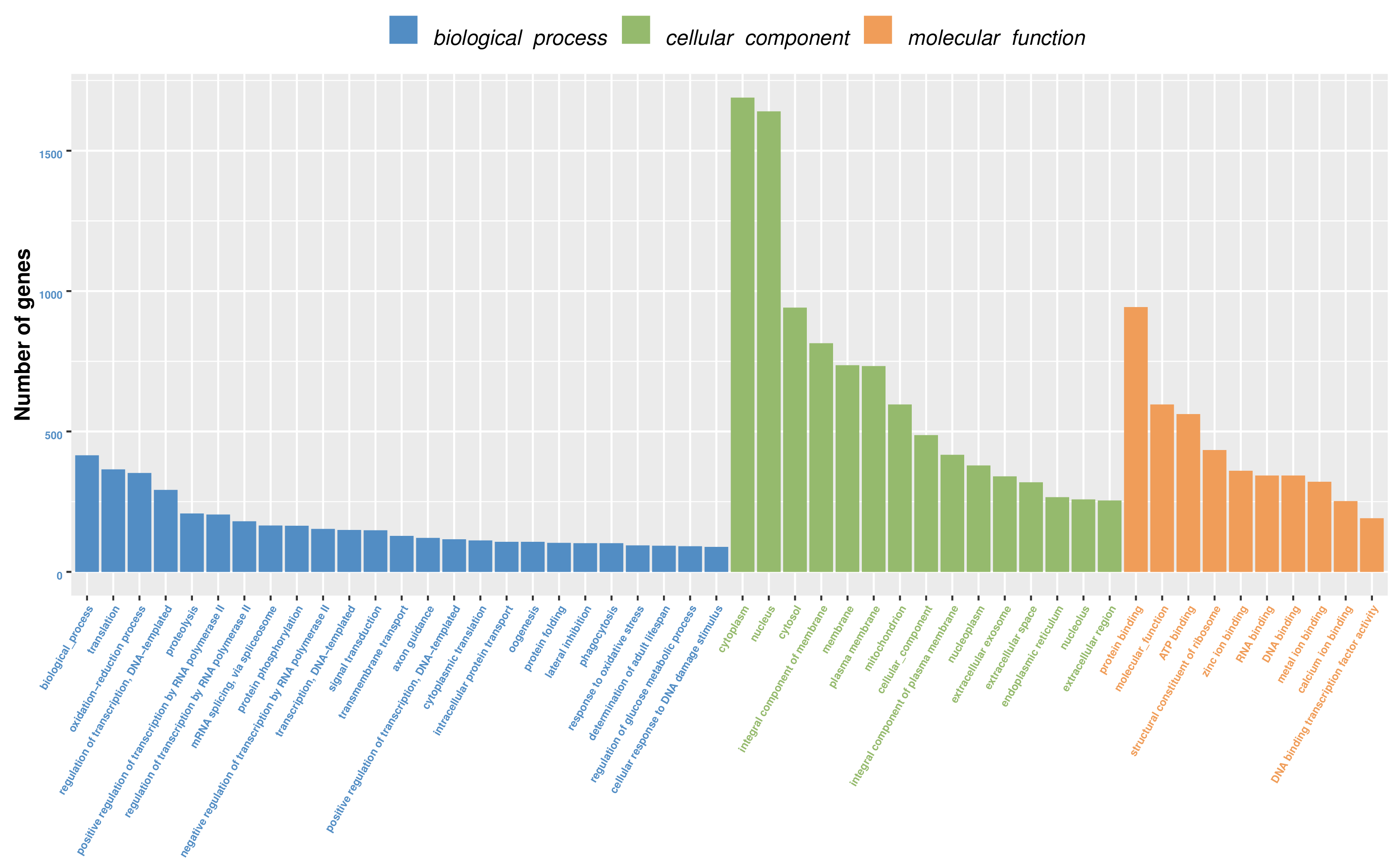

Supplement: Supplementary Figure S2 — Gene Ontology (GO) annotation and classification of the A. craccivora. [file Image_2.tif]

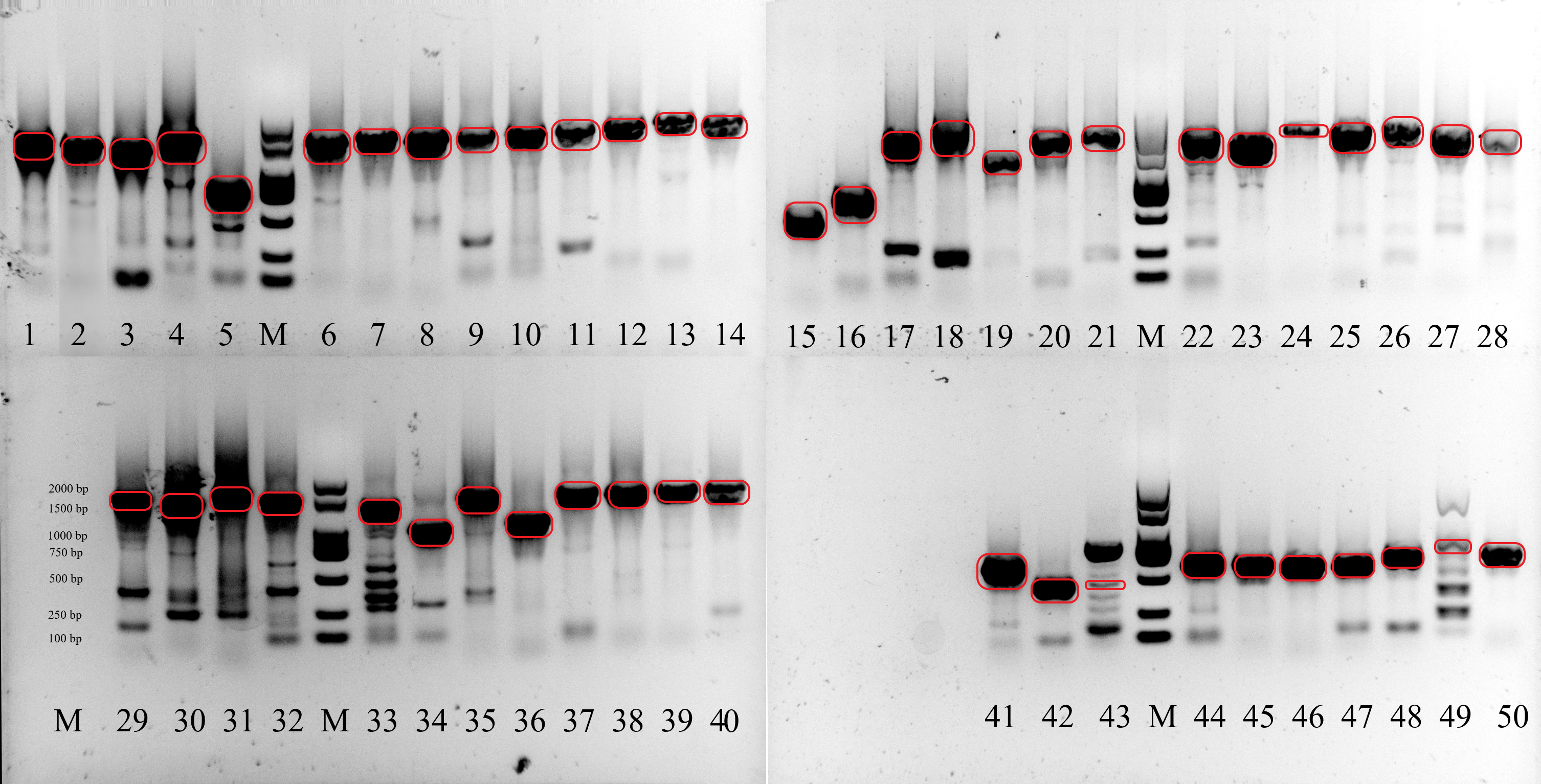

Supplement: Supplementary Figure S3 — Electrophoretic images of 50 genes. M: marker, the marker from top to bottom are 2,000 bp, 1,500 bp, 1,000 bp, 750 bp, 250 bp, and 100 pp. 1, CYP301B1; 2, CYP315A1; 3, CYP353B1; 4, CYP6CY22; 5, CYP6YC1; 6, CYP18A1; 7, CYP6DB1; 8, CYP306A1; 9, CYP6CY22; 10, CYP6CY7; 11, CYP4CJ2; 12, CYP6CY52; 13, CYP380C6; 14, CYP4CJ1; 15, GSTM1; 16, GSTD1; 17, CYP6CY21; 18, CYP6CY8; 19, CYP6CY5; 20, CYP6CY48; 21, CYP4CJ14; 22, CYP4CJ5; 23, CYP6DA1; 24, CYP6DA2; 25, CYP6CY14; 26, CYP6CZ1; 27, CYP314A1; 28, CYP6DC1; 29, CYP301A1; 30, CYP305E1; 31, CYP4G51; 32, CYP6CY13; 33, CYP307A2; 34, CYP302A1; 35, CYP6DD1; 36, CYP303A1; 37, CYP4CH1; 38, CYP4CK1; 39, CYP4CJ13; 40, CYP6CY56; 41, RPL11; 42, RPS8; 43, GSTM2; 44, CYPGSTD2; 45, GSTS1; 46, GSTS3; 47, GSTS2; 48, GSTT2; 49, GSTO1; 50, GSTT1. [file Image_3.tiff]
